# Spatial distribution of marker compounds in *Epimedium sagittatum* leaves and the role of UGT72B113 in generating kaempferol glycoside diversity

**DOI:** 10.1093/hr/uhag187

**Published:** 2026-05-09

**Authors:** Bin Yan, Xinghao Zhang, Ziyan Xie, Huanhuan Liang, Yongxia Jia, Hanxiang Li, Yuping Li, Yongqing Li, Junjie Wei, Ying Wang, Xiaoman Yang

**Affiliations:** State Key Laboratory of Plant Diversity and Specialty Crops, Guangdong Provincial Key Laboratory of Applied Botany, Guangdong Provincial Key Laboratory of Digital Botanical Garden, South China Botanical Garden, Chinese Academy of Sciences, Guangzhou 510650, China; College of Life Sciences, University of Chinese Academy of Sciences, Beijing 100049, China; State Key Laboratory of Plant Diversity and Specialty Crops, Guangdong Provincial Key Laboratory of Applied Botany, Guangdong Provincial Key Laboratory of Digital Botanical Garden, South China Botanical Garden, Chinese Academy of Sciences, Guangzhou 510650, China; College of Life Sciences, University of Chinese Academy of Sciences, Beijing 100049, China; Key Laboratory of National Forestry and Grassland Administration on Chinese Herbal Medicine, College of Life Science, Northeast Forestry University, Harbin 150040, China; College of Agronomy, Henan Agricultural University, Zhengzhou 450046, China; State Key Laboratory of Plant Diversity and Specialty Crops, Guangdong Provincial Key Laboratory of Applied Botany, Guangdong Provincial Key Laboratory of Digital Botanical Garden, South China Botanical Garden, Chinese Academy of Sciences, Guangzhou 510650, China; State Key Laboratory of Plant Diversity and Specialty Crops, Guangdong Provincial Key Laboratory of Applied Botany, Guangdong Provincial Key Laboratory of Digital Botanical Garden, South China Botanical Garden, Chinese Academy of Sciences, Guangzhou 510650, China; State Key Laboratory of Plant Diversity and Specialty Crops, Guangdong Provincial Key Laboratory of Applied Botany, Guangdong Provincial Key Laboratory of Digital Botanical Garden, South China Botanical Garden, Chinese Academy of Sciences, Guangzhou 510650, China; College of Life Sciences, University of Chinese Academy of Sciences, Beijing 100049, China; State Key Laboratory of Plant Diversity and Specialty Crops, Guangdong Provincial Key Laboratory of Applied Botany, Guangdong Provincial Key Laboratory of Digital Botanical Garden, South China Botanical Garden, Chinese Academy of Sciences, Guangzhou 510650, China; College of Life Sciences, University of Chinese Academy of Sciences, Beijing 100049, China; College of Agronomy, Henan Agricultural University, Zhengzhou 450046, China; State Key Laboratory of Plant Diversity and Specialty Crops, Guangdong Provincial Key Laboratory of Applied Botany, Guangdong Provincial Key Laboratory of Digital Botanical Garden, South China Botanical Garden, Chinese Academy of Sciences, Guangzhou 510650, China; College of Life Sciences, University of Chinese Academy of Sciences, Beijing 100049, China; State Key Laboratory of Plant Diversity and Specialty Crops, Guangdong Provincial Key Laboratory of Applied Botany, Guangdong Provincial Key Laboratory of Digital Botanical Garden, South China Botanical Garden, Chinese Academy of Sciences, Guangzhou 510650, China; College of Life Sciences, University of Chinese Academy of Sciences, Beijing 100049, China

## Abstract

*Epimedium sagittatum* is an important medicinal plant whose main bioactive flavonol glycosides, such as epimedin C and icariin, are derived from kaempferol. However, the spatial distribution of these compounds within the plant and the genetic basis for their structural diversity remain unclear. Here, we employed MALDI-TOF mass spectrometry imaging to visualize the distribution of epimedin C and icariin in *E. sagittatum* leaves, revealing their predominant accumulation in nonleaf vein mesophyll part. Integrated metabolomic and transcriptomic analyses of leaves, petioles, and rhizomes highlighted glycosylation as a key contributor to kaempferol derivative diversity. We identified five candidate UDP-glycosyltransferase genes, among which UGT72B113 exhibited remarkable functional versatility. *In vitro* enzymatic assays demonstrated that UGT72B113 accepts multiple kaempferol-type aglycones and various sugar donors, catalyzing the formation of monoglycosylate, diglycosylate, and even triglycosylated products from a single substrate. Our findings reveal that UGT72B113 is involved in generating flavonol glycoside diversity in *E. sagittatum* via its broad substrate promiscuity, multi-glycosylation capability, and sugar donor flexibility. This study provides new insights into the spatial regulation of flavonol accumulation and the enzymatic mechanisms underlying phytochemical diversity in medicinal plant.

## Introduction

Flavonoids are widely distributed throughout the plant kingdom, where they serve essential roles in protecting plants from diverse environmental stresses [1]. For humans, flavonoidsexhibit extensive pharmacological benefits and have been utilized as therapeutic agents and functional food ingredients [2]. Structural diversification of flavonoids arises from different modifications of the conserved C6–C3–C6 backbone. It includes glycosylation, methylation, acylation, prenylation, and so on. These modifications generate substantial chemical variation and also influence the structure–activity relationships. For instance, glycosylation and acylation enhance the stability of anthocyanins and broaden their application in food industries [3]. Prenylation increases lipophilicity, leading to higher affinity for cell membranes and thereby augmenting bioactivities like antibacterial and anti-inflammatory properties [4]. Thus, the diversity of the flavonoids is a key determinant of their varied biological functions [5].

Glycosylation, a key driver of this diversity, is primarily catalyzed by UDP-dependent glycosyltransferases (UGTs). Numerous UGTs have been reported to function in flavonoid biosynthesis [6]. They mediate both anabolic and catabolic processes, contributing to compound stability, solubility, and the formation of structurally diverse glycosides [5, 7, 8]. UGTs constitute one of the largest plant gene families, with dozens to hundreds of members identified per species. For example, there are 409 UGTs in *Medicago sativa* [9] and 339 in *Epimedium pubescens* [10]. The remarkable diversity of plant flavonoid glycosides can be attributed to UGT gene expansion [11] and functional differentiation [12]. Despite progress in characterizing UGT families, it remains unclear how specific UGTs coordinate to shape the glycoside richness observed in some medicinal plants.

*Epimedium* (Berberidaceae) is a renowned worldwide as a garden plant for its attractive flowers, elegant foliage, and tolerance to shade and drought [13]. In Asia, particularly China, Japan, and Korea, it has a long history of medicinal use. Pharmacological studies have demonstrated its osteoprotective [14, 15], sexual enhancement [15], antitumor [16], and neuroprotective properties [17]. Flavonoids represent the major bioactive constituents [18]. The Chinese Pharmacopoeia specifies epimedin A, B, C, and icariin as marker compounds. These molecules share a kaempferol flavonol core but differ in glycosylation patterns. Among the 274 chemical components reported from this genus, 199 are flavonoids, the majority of which exist in glycosylated forms [18]. It underscores glycosylation as a central theme in its phytochemistry.

Significant interspecific chemical variation exists with *Epimedium* [19]. *Epimedium sagittatum* is one of the most widely distributed species in China, exhibiting considerable morphological variation and marked divergence in functional components across geographical populations [20]. These characteristics make it an ideal species for both traditional herbal use and the extraction of bioactive compounds. However, a systematic understanding of its flavonoid profile, particularly the distribution of specific marker compounds and the genetic basis of flavonoid glycoside diversity remain poorly understood.

Therefore, this study focuses on *E. sagittatum* to first elucidate the distribution of its marker flavonoid compounds by matrix-assisted laser desorption/ionization-time of flight mass spectrometry imaging (MALDI-TOF MSI). We then employ a combined metabolomic and transcriptomic approach to comprehensively profile its flavonoids, with particular emphasis on kaempferol derivative, and to investigate the associated biosynthetic pathway. Our investigation suggests that UGT72B113 serve as a candidate factor involving in the formation of kaempferol derivatives. Our findings will deepen the understanding of how UGT generates flavonoid glycoside diversity and identify potential target genes for molecular breeding or the biosynthesis of valuable flavonoid glycosides.

## Results

### Distribution of marker compounds in the leaf of *E. sagittatum*

Epimedin A, B, C, and icariin are designated as marker compounds of *E. sagittatum* in the Chinese Pharmacopoeia. Given that the leaves serve as the medicinal part of *E. sagittatum*, the distribution pattern of these marker compounds within the leaf has not been previously explored. Therefore, this study employed MALDI-TOF MSI to investigate their spatial distribution.

To determine the optimal MALDI conditions for simultaneous detection of the marker compounds in leaf tissue, we used a mixture of four authentic standards, including epimedin A, epimedin B, epimedin C, and icariin as a sample. Four matrixes including α-cyano-4-hydroxycinnamic acid (CHCA), sinapic acid (SA), 9-aminoacridine (9-AA), and 1,5-diaminonaphthalene (1,5-DAN) were used. Under the tested conditions, none of the four compounds could be detected simultaneously with CHCA, SA, or 9-AA (Supplementary Figs S1–S6). Simultaneous detection of all four authentic standards was achieved only with 1,5-DAN in positive ion mode (Supplementary Fig. S7). Consequently, 1,5-DAN was selected as the matrix for all subsequent imaging experiments.

**Figure 1:**
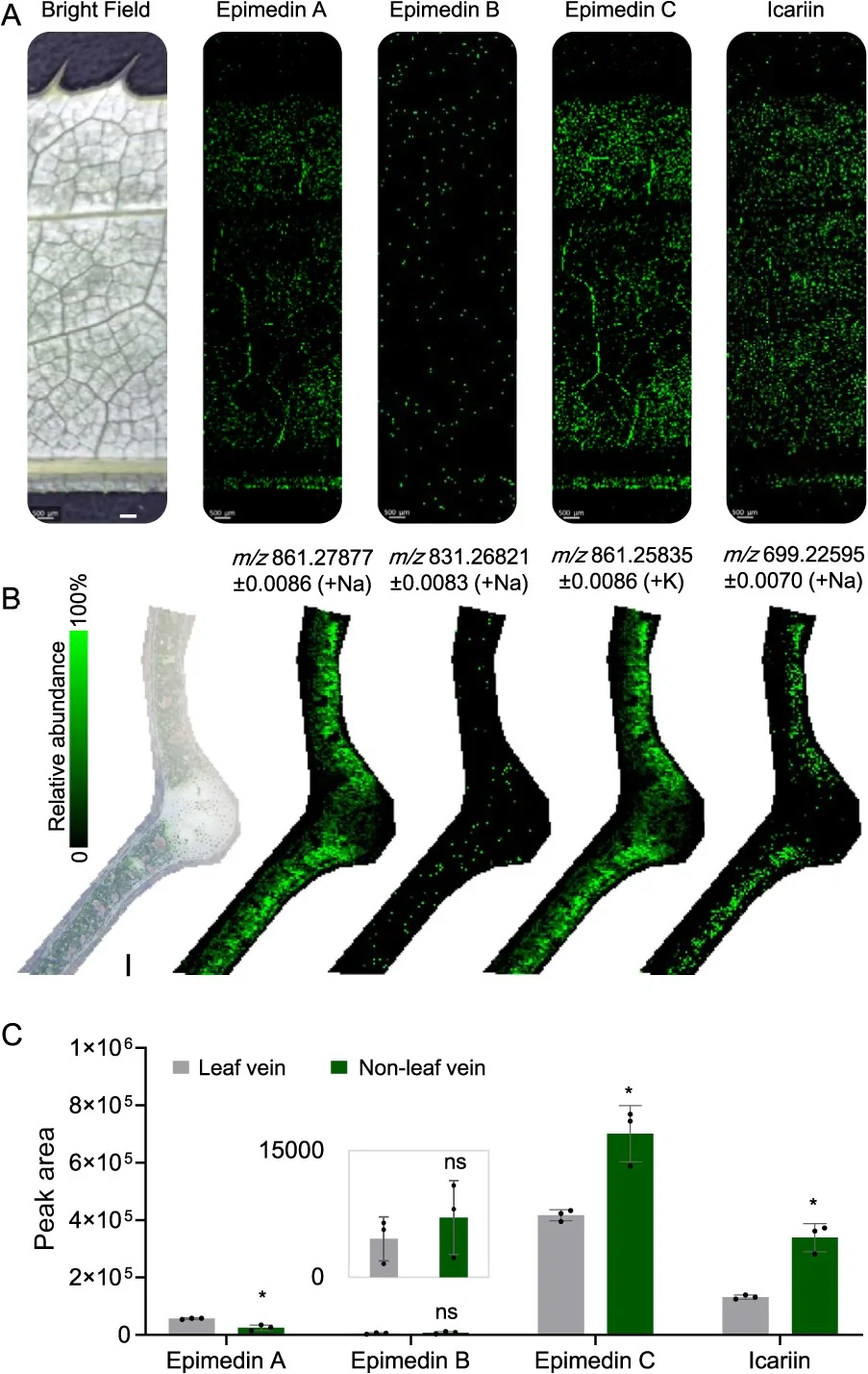
Distribution of marker compounds in *E. sagittatum* leaves. (A) Extracted spatial distribution image of epimedin A, B, C, and icariin on the adaxial leaf surface. Acquired at 50 × 50 μm pixel size. Mass tolerance was set to 10 ppm. Scale bar = 500 μm. (B) Distribution of the same four compounds across leaf cross-section, including the main vein and non-vein regions. Acquired at 10 × 10 μm pixel size. Mass tolerance was set to 10 ppm. Scale bar = 100 μm. The absolute *m/z* and ion adduct forms of each compounds are indicated. (C) Relative quantification of the four marker compounds by HPLC. A magnified view of the epimedin B result is shown in the inset. Data are shown as mean ± *SD* (*n* = 3 biological replicates, the black dots represent each individual samples). Statistical significance was assessed using Student’s *t*-test using Microsoft Excel: ns, not significant (*P* > 0.05); ^*^, *P* ≤ 0.05.

Due to the large size of the *E. sagittatum* leaf, we selected part of the leaf as a sample. Under the optimized conditions, we extracted the spatial distribution of the four marker compounds with *m/z* of 861.27877 ± 0.0086, 831.26821 ± 0.0083, 861.25835 ± 0.0086, and 699.22595 ± 0.0070 in the MS image (Fig. 1A). Based on the theoretical *m/z* values of the four marker compounds, these *m/z* values were assigned to the [M + Na]^+^ ions of epimedin A, [M + Na]^+^ ions of epimedin B, [M + K]^+^ ions of epimedin C, and [M + Na]^+^ ions of icariin, respectively. Many other molecular weights were also detected, but they could not be assigned to specific compounds due to the lack of authentic standards.

According to the relative signal intensities of each compound, epimedin B exhibited relatively lower abundance compared to the other three compounds (Fig. 1A and B). All four compounds were uniformly distributed across the leaf, but some differences were observed between the main leaf vein and non-leaf vein regions. Specifically, the compounds were undetectable in the main vein of the leaf (Fig. 1A), suggesting heterogeneous distribution patterns of these four compounds between the leaf vein and non-leaf vein areas.

To further investigate this distribution, we prepared a cross-section of the leaf that included both main leaf vein and non-leaf vein regions (Fig. 1B). Consistent with Fig. 1A results, epimedin B remained the least abundant compound in the leaf. Epimedin A, C, and icariin were predominantly distributed in the nonleaf vein regions, with minimal presence in the main leaf vein (Fig. 1B).

**Figure 2:**
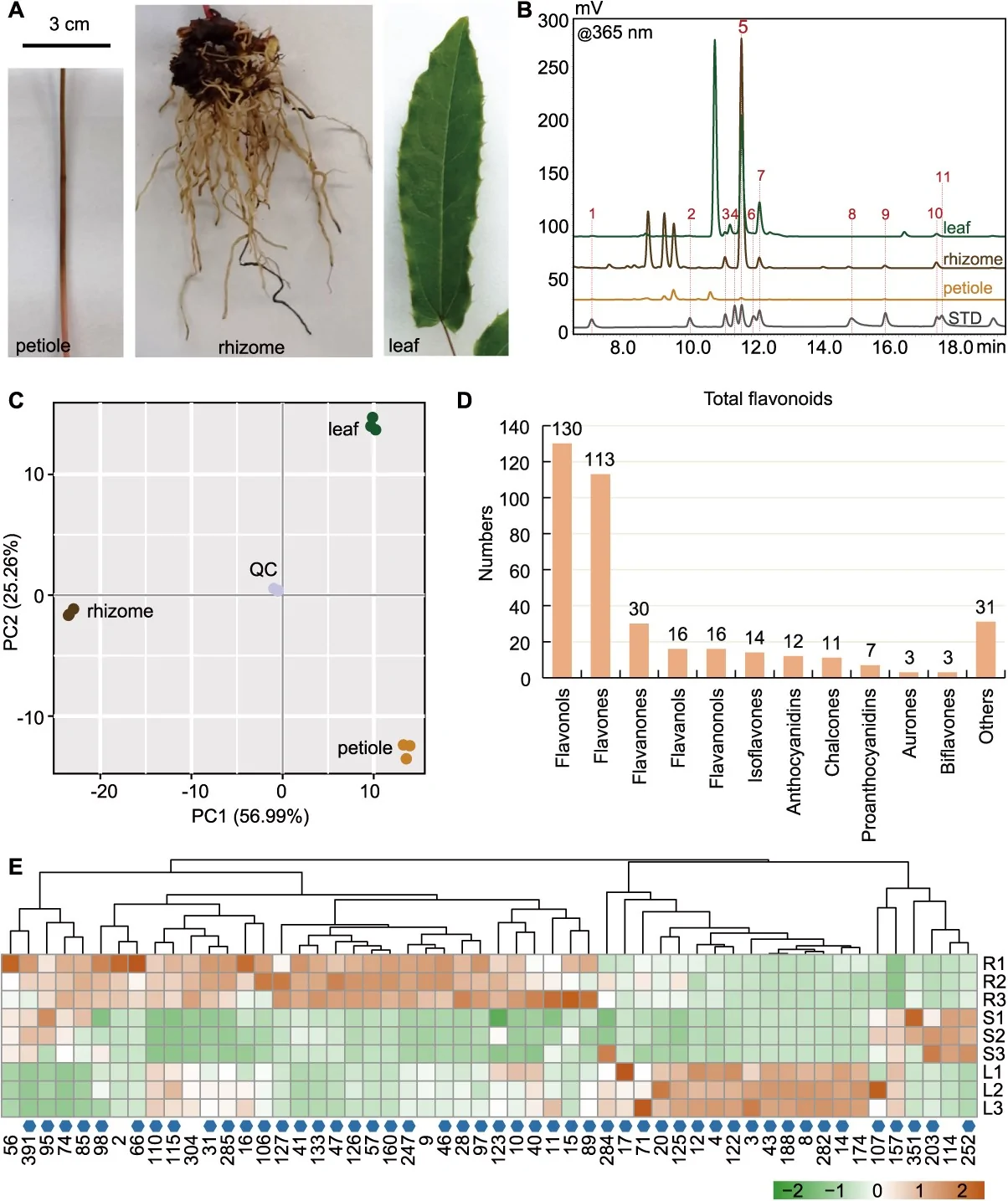
Metabolite profiling of *E. sagittatum*. (A) The three plant parts used in this study. (B) HPLC chromatograms of extracts from each part. Peaks are labeled as: 1, Epimedoside A; 2, Afzelin; 3, Epimedin A; 4, Epimedin B; 5, Epimedin C; 6, Kaempferol-7-*O*-rhamnoside; 7, Icariin; 8, Kaempferol; 9, Baohuoside II; 10, 2′′-*O*-rhaminosyl icariside II; 11, Icariside I. (C) Principal component analysis (PCA) of metabolomic data from the three parts. (D) Counts of tentatively identified flavonoids in each part. (E) Hierarchical clustering analysis of kaempferol derivatives based on peak areas (Supplementary Table S1). Glycosylated compounds are marked with a blue hexagon. Three biological replicates were prepared for each sample.

To validate these observations, we separated the main leaf veins and the remaining parts and measured the relative contents of the four compounds using peak areas as relative content. The results indicated that both epimedin C and icariin had lower concentrations in the main leaf veins compared to the other part (Fig. 1C), which corroborated the MSI results. For epimedin B, its low abundance in the tissue precluded reliable detection, making a direct comparison infeasible. Epimedin A showed a discrepancy between the spatial distribution observed by MSI and the relative quantitative High-Performance Liquid Chromatography (HPLC) results (Fig. 1). This inconsistency may be explained by the relatively low endogenous levels of epimedin A. Low compound abundance in planta increases susceptibility to signal interference and background noise, which can compromise detection accuracy. Indeed, among the four markers, epimedin C and icariin were the most abundant in our samples, whereas epimedin A and epimedin B were present at substantially lower levels (Fig. 2B). Collectively, these findings indicate that only the distribution patterns of epimedin C and icariin are reliably supported by quantitative validation, underscoring the importance of complementing MSI with tissue-dissected HPLC analysis.

### Accumulation of flavonoids in the rhizomes, petioles, and leaves of *E. sagittatum*

The four marker compounds belong to flavonoids. In this section, we delve deeper into the details of flavonoid accumulation. To investigate the distribution patterns of flavonoids from *E. sagittatum* across different tissues including rhizomes, petioles, and leaves (Fig. 2A), an HPLC analysis was firstly performed to examine the chemical fingerprints of three parts. Some commercial standards were used to identify the compounds present in the extract.

As illustrated in Fig. 2B, the leaves, rhizomes, and petiole exhibited distinct chemical fingerprints. Judged by the peak height, epimedin C is the most abundant marker compound in the leaves of tested *E. sagittatum*, followed by icariin, epimedin A, and epimedin B. This result is consistent with Fig. 1. When comparing across different tissues, epimedin C showed relatively higher concentrations in the leaves and rhizomes but was notably lower in the petioles. The content of icariin is highest in the leaves, followed by rhizome, and lowest in the petiole. Epimedin A was only detected in the leaves and rhizomes, but not in the petioles. The content of epimedin B was the lowest, with only trace amounts present in all three parts, exhibiting extremely low peak heights (Fig. 2B). Additionally, other kaempferol derivatives, for which commercial standards were available, were also detected but did not exhibit high concentrations in any of the three parts.

To further elucidate the detailed differences in flavonoid profiling, a nontargeted flavonoid metabolomics analysis was performed. A total of 395 flavonoids were tentatively identified (Supplementary Table S1). Principal component analysis (PCA) using all these compounds revealed that the leaves, rhizomes, and petioles could be clearly distinguished by PC1 (accounting for 56.99% of the variance) and PC2 (accounting for 25.26% of the variance) (Fig. 2C). This result indicates significant differences among the three parts, which is consistent with the HPLC fingerprint results (Fig. 2B).

Among the 395 tentatively identified flavonoids, they were further classified into different classes, including flavonols, flavones, flavanones, and others. Among them, 130 compounds belonged to flavonols, while 113 compounds were classified as flavones. The remaining categories contained fewer than 30 compounds each (Fig. 2D). This distribution highlights that flavonols are the most prominent group of flavonoids in *E. sagittatum*.

Within the 130 flavonols, 54 were identified as kaempferol derivatives, with the remainder belonging to other flavonols. The distribution of these 54 compounds varied among the three parts. According to the heat map with relative content of these compounds, the rhizome part contained the highest diversity of kaempferol derivatives, followed by the leaves, with the petioles showing the least diversity (Fig. 2E). Further structural investigation of these 54 kaempferol derivatives revealed that 48 out of 54 (~88.89%) were modified through glycosylation, featuring one or several glycosyl substitutions. Glucosyl and rhamnosyl moieties were the most frequently observed modifications (Supplementary Table S1). These findings suggest that glycosylation plays a crucial role in the diversity of kaempferol derivatives in *E. sagittatum*.

### Identification of differentially expressed genes and pathway enrichment

Since different parts of a plant share the same genome, variations in metabolite profiles primarily from differences in gene expression. Investigating gene expression patterns across distinct plant parts can further elucidate the molecular mechanisms underlying metabolite biosynthesis. To this end, transcriptomic sequencing analysis on samples from the leaves, rhizomes, and petioles of *E. sagittatum* was conducted to identify gene expression patterns.

**Figure 3:**
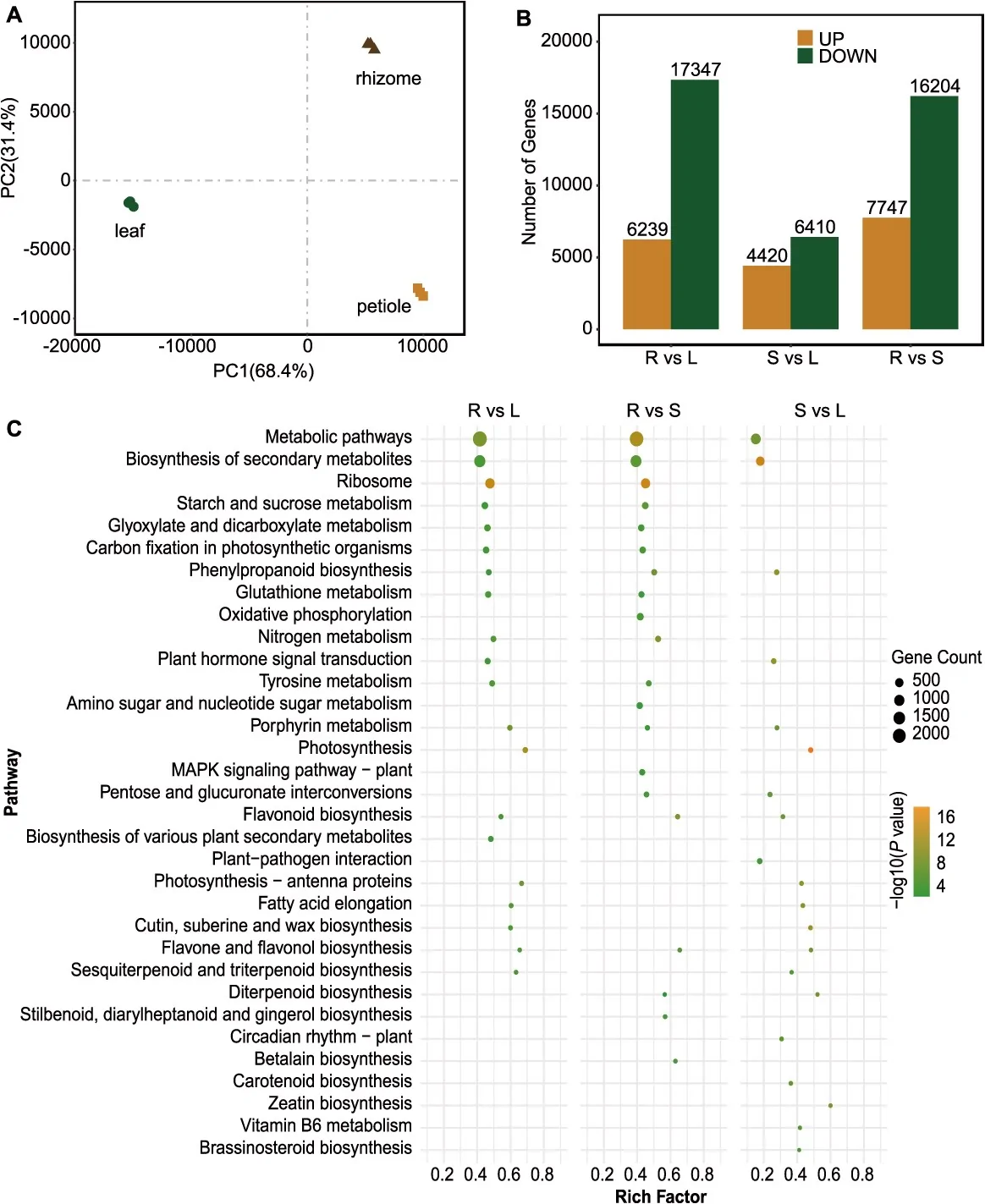
Differential gene expression and enrichment analysis. (A) PCA of gene expression profiles across all three samples. (B) Numbers of upregulated and downregulated genes in each pairwise comparison (R, rhizomes; S, petioles; L, leaves). (C) Pathway enrichment analysis of DEGs for each comparison. Only pathways with *P* ≤ 0.05 are displayed. Node color gradient orange to green indicates *P* value high to low, node size reflects the number of DEGs in the pathway, and labels show gene count and rich factor (proportion of DEGs in the pathway). The top 20 enriched pathways per comparison are shown.

A total of 67.99 billion base pairs of reads were obtained from the RNA-seq dataset encompassing nine samples. Each sample yielded an average of 7.55 Gb of data, with an average Q20 score of approximately 97.35% and each individual score exceeding 96% (Supplementary Table S2), indicating the high quality of the transcriptomic sequencing and assembly. PCA results revealed that the samples from leaves, rhizomes, and petiole were clearly separated, with PC1 accounting for 68.4% and PC2 for 31.4% of the variance, indicating significant differences in gene expression among samples from three parts (Fig. 3A).

Among these samples, the number of differentially expressed genes (DEGs) in the rhizome versus leaf (R vs L) comparison group was 23 586, with 17 347 downregulated and 6239 upregulated. In the petiole versus leaf (S vs L) comparison, there were 10 830 DEGs, with 6410 downregulated and 4420 upregulated. The rhizome versus petiole (R vs S) comparison yielded 23 951 DEGs, with 16 204 downregulated and 7747 upregulated (Fig. 3B). The transcriptomic data and the metabolites results exhibited similar patterns, indicating significant differences among different parts of *E. sagittatum* plants.

Furthermore, KEGG pathway enrichment analysis was performed on the DEGs, and the top 20 pathways based on gene count enrichment were presented (Fig. 3C). The DEGs in the R vs L, R vs S, and S vs L comparison groups were enriched in the pathways, such as metabolic pathways, biosynthesis of secondary metabolites, ribosome, and others. These enriched pathways of DEGs may collectively contribute to the variations in metabolite content across different parts of *E. sagittatum* plants. Specifically, among the top 20 enriched pathways, all three comparison groups exhibited differences involving flavone and flavonol biosynthesis (Fig. 3C). This suggests that genes involved in the flavonol pathway play a significant role in the flavonol content variations observed among the three parts of *E. sagittatum*.

### Outline of kaempferol derivatives biosynthesis in *E. sagittatum*

Flavonols, particularly kaempferol derivatives, are the most critical compounds for the utilization of *Epimedium* plants [18]. Following the identification of a substantial number of glycosylated flavonols in the metabolome and the enrichment of the flavonol pathway in the KEGG enrichment analysis, this section aims to further elucidate the pathway genes involved in biosynthesis of kaempferol derivatives.

**Figure 4:**
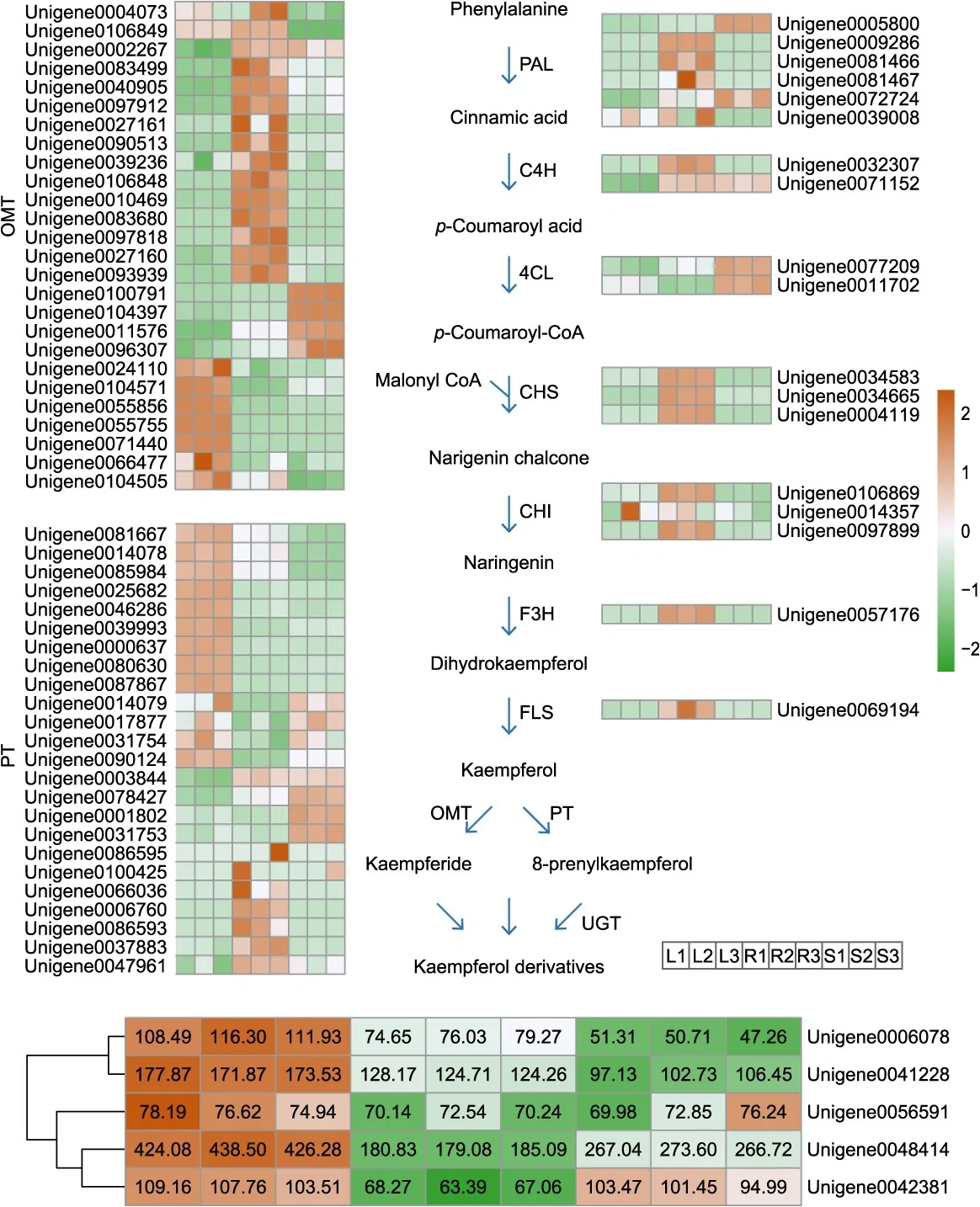
Expression of genes involved in kaempferol derivative biosynthesis. RPKM values are represented by a color scale (brick red = high, green = low). L: leaf, R: rhizome, S: petiole.

As shown in Fig. 4, pathway genes exhibit distinct expression patterns across the leaves, rhizomes, and petioles. Starting from phenylalanine, genes involved in the early stages of kaempferol biosynthesis, such as phenylalanine ammonia-lyase (PAL), cinnamate 4-hydroxylase (C4H), chalcone synthase (CHS), chalcone isomerase (CHI), flavanone 3-hydroxylase (F3H), and flavonol synthase (FLS), predominantly show high expression levels in the rhizomes. In contrast, 4-coumarate: CoA ligase (4CL) genes exhibited relatively higher expression in both the petioles and rhizomes. Furthermore, we selected several genes from this pathway and performed quantitative reverse transcription-polymerase chain reaction (qRT-PCR) analysis. The calculated relative expression levels of these genes across the three tissues (Supplementary Fig. S8) were consistent with the trends observed in the transcriptome data (Fig. 4), confirming the reliability of the RNA-seq results.

Following the synthesis of kaempferol, several modifications occur to generate diverse derivatives, such as prenylation, *O*-methylation, and glycosylation. From the RNA-seq data, 26 *O*-methyltransferases (OMTs) and 23 prenyltransferases (PTs) have been identified. These genes displayed distinct expression patterns. Among the OMTs, most selected genes exhibit high expression levels in the rhizomes, followed by the leaves, and are least expressed in the petioles. Among the PTs, selected genes are predominantly expressed in the leaves, followed by the petioles and rhizomes (Fig. 4).

Given that over 88% of kaempferol derivatives are glycosylated, we further focused on UGTs as key contributors to the diversity of kaempferol glycosides. A total of 96 genes from the UGT family were screened (Supplementary Fig. S9). Considering that kaempferol glycosides are generally accumulated throughout the plant, including the leaves, rhizomes, and petioles, this study hypothesized that UGT genes highly expressed across all three parts might contribute to the diversity of kaempferol glycosides. After normalizing gene expression levels, we selected five genes that exhibited high expression across all three parts: *unigene0006078*, *unigene0041228*, *unigene0056591*, *unigene0048414*, and *unigene0042381* (Supplementary Fig. S9). All five candidate genes displayed RPKM (Reads Per Kilobase Million) values exceeding 50, with the highest exceeding 400 (Supplementary Table S3). The expression patterns of these genes were further analyzed using a heat map, all five genes expressed higher in the leaves than the petioles and rhizomes (Fig. 4).

These candidate genes were renamed with the help of UNCC (UGT Nomenclature Committee) based on their protein sequences. Specifically, *unigene0006078* was renamed *UGT71CE1*, *unigene0041228* as *UGT85A213*, *unigene0056591* as *UGT84AA1*, *unigene0048414* as *UGT72B113*, and *unigene0042381* as *UGT74DV1*, respectively.

### Functional characterization of candidate UGTs from *E. sagittatum*

To validate the functions of the candidate UGT genes in the glycosylation of kaempferol derivatives, full-length coding regions of these candidate genes were cloned from the cDNA. Then, recombinant proteins fused with a His_6_ tag were induced. SDS-PAGE analysis was employed to confirm the successful expression of these proteins (Supplementary Fig. S10).

**Figure 5:**
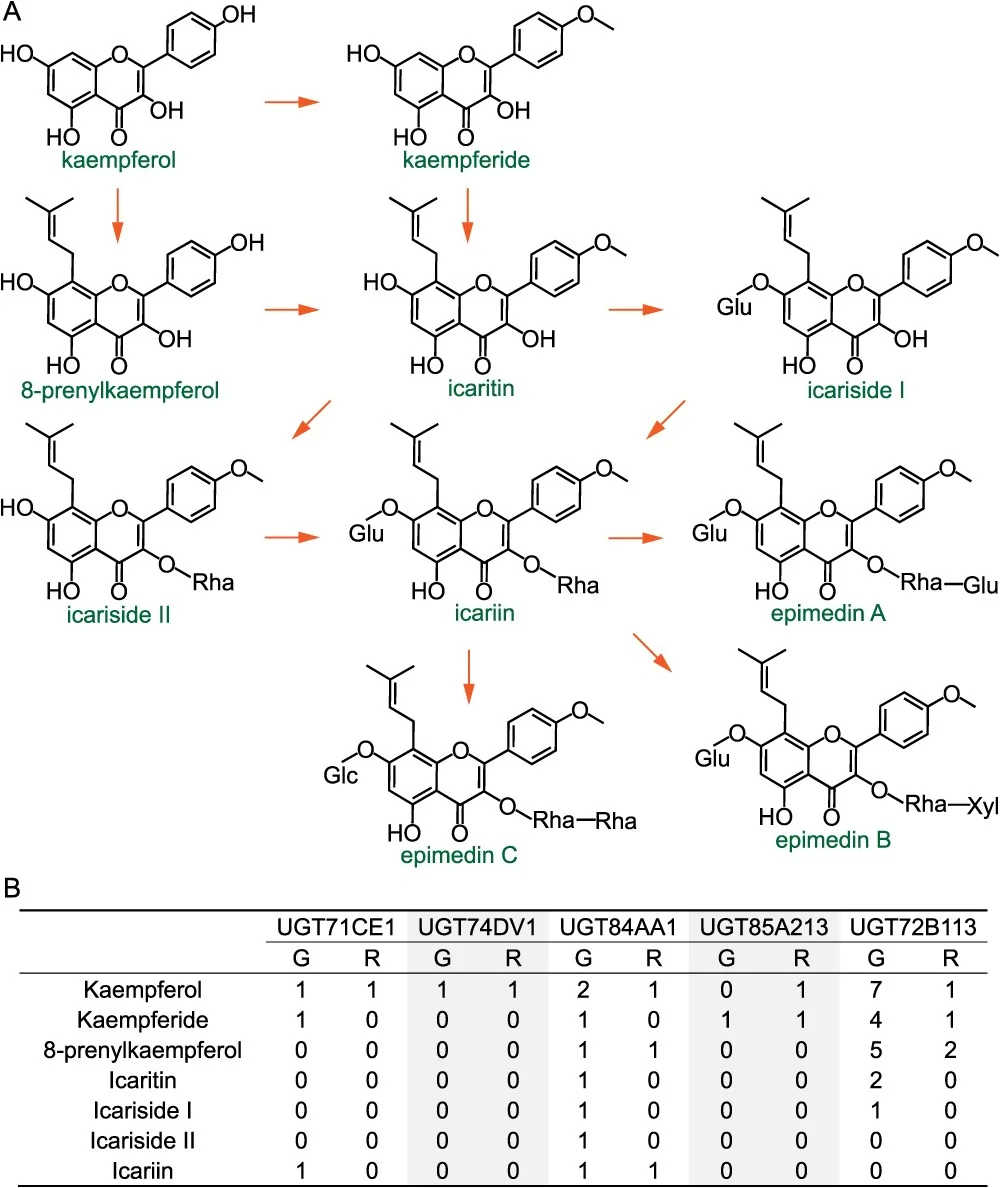
Compound structure diversity from kaempferol to four marker compounds. (A) Biosynthesis pathway of epimedin A, B, C, and icariin from kaempferol. (B) Number of products detected during crude enzyme screening with different UDP-sugar donors: G, UDP-glucose; R, UDP-rhamnose.

Next, crude enzyme extracts were used to screen for their activity towards various kaempferol derivatives from the icariin pathway (Fig. 5A). Given that glucosyl and rhamnosyl moieties were the most prevalent in these derivatives (Supplementary Table S1), UDP-glucose and UDP-rhamnose were selected as sugar donors to assess the crude enzyme activity of the recombinant proteins.

The enzymatic reactions were analyzed using HPLC. The tentative product numbers identified in the reactions were summarized (Fig. 5B, Supplementary Figs S11–S13). Based on the HPLC results, we found that the recombinant UGT71CE1 could catalyze the glycosylation of kaempferide using both UDP-glucose and UDP-rhamnose as sugar donors, generating a single product respectively. Additionally, it could $$catalyze the substrate kaempferide and icariin when UDP-glucose was used as sugar donor. UGT74DV1 could only catalyzed kaempferol with UDP-glucose and UDP-rhamnose as sugar donors. UGT84AA1 exhibited a broader substrate promiscuity, accepting all of the tested substrates when using UDP-glucose as sugar donor, when UDP-rhamnose was added, kaempferol, 8-prenylkaempferol, and icariin were also can be transferred to glycosylated products. For UGT85A213, the substrate kaempferol was accepted when incubating with UDP-rhamnose and kaempferide was accepted with both sugar donors (Fig. 5B).

**Figure 6:**
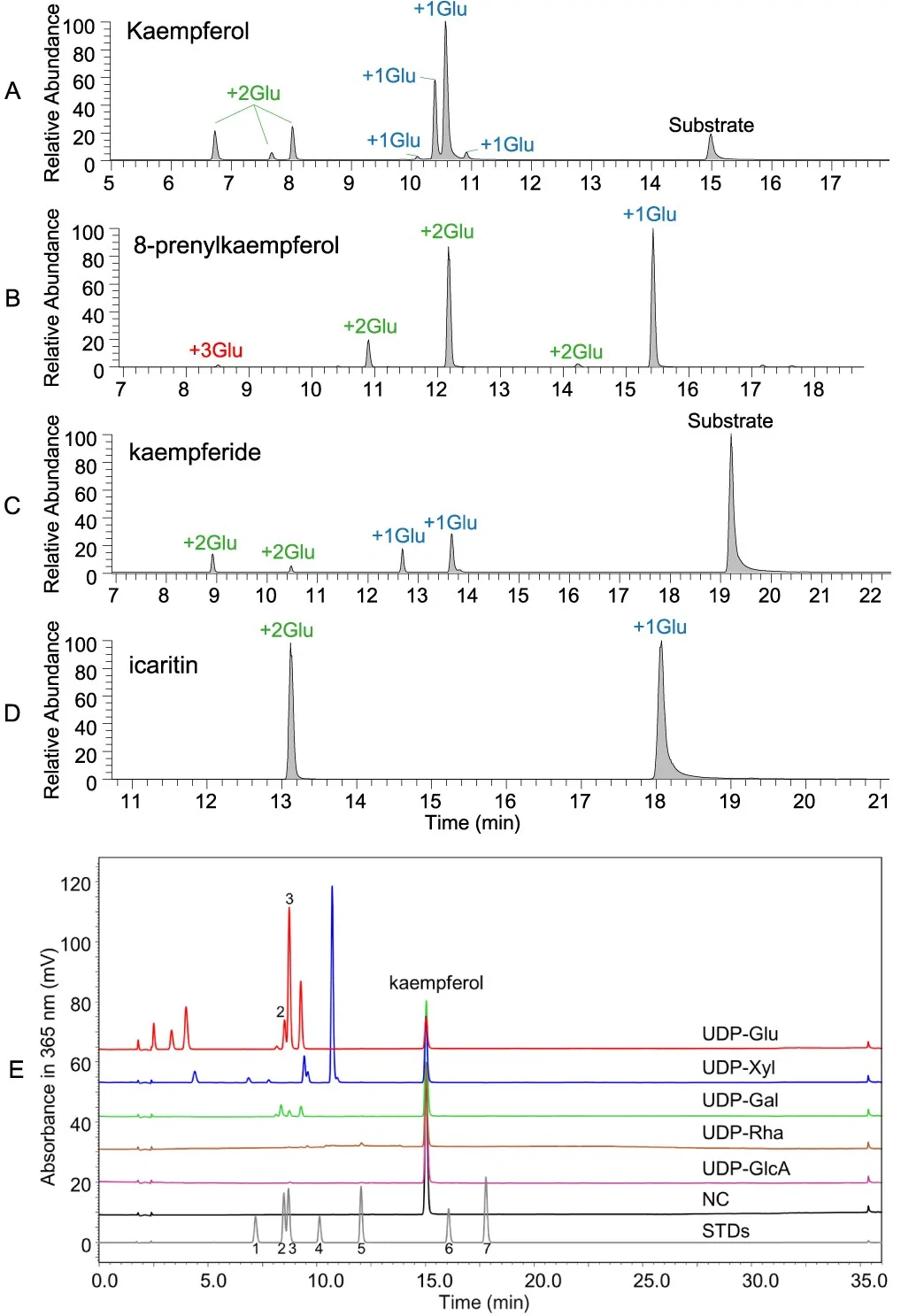
Product diversity of UGT72B113. (A–D) UHPLC–MS/MS analysis of glycosylated products. The number of glycosyl moiety was labeled. (E) HPLC detection of UGT72B113 activity using kaempferol as substrate with different UDP-sugar donors. Standards 1: kaefmperol-3-*O*-rutinoside, 2: kaempferol-3-*O*-glucoside, 3: kaempferol-7-*O*-glucoside, 4: kaempferitrin, 5: kaempferol-7-rhamnoside, 6: baohuoside II, 7: icariside I.

Notably, UGT72B113 emerged as the most reactive enzyme. The recombinant UGT72B113 proteins were capable of catalyzing most of the tested substrates except icariside II and icariin. This enzyme displayed a unique property compared to the others. The most distinct result was that there are more than two products when using kaempferol, kaempferide, 8-prenylkaempferol, and icaritin as substrates and UDP-glucose as the sugar donor (Figs 5B and 6A). The versatility of UGT72B113 in catalyzing multiple kaempferol derivatives and producing diverse products from a single substrate indicates its important role in generating flavonol diversity in *E. sagittatum*.

The enzymatic kinetic parameters of UGT72B113 were determined using these four compounds as substrate. Due to the low concentration of the products generated when icaritin was used as the substrate, no reliable kinetic data could be obtained for this compound. Among the other three substrates, kaempferide exhibited the lowest *K*_m_ value (4.51 ± 0.76 μmol·l^−1^), followed by kaempferol (17.64 ± 1.63 μmol·l^−1^) and 8-prenylkaempferol (26.80 ± 6.26 μmol·l^−1^) (Supplementary Fig. S14), indicating that kaempferide is the most preferred substrate for UGT72B113.

### Glycosyl diversity caused by UGT72B113

From the aforementioned results, we can infer that UGT72B113's ability to accept different flavonol substrates is one of the key factors contributing to flavonol diversity in *E. sagittatum*. Furthermore, our study would reveal that UGT72B113 is also responsible for generating multiple glycosyl products of flavonols. These would be elucidated by the identification of the structures of the products. We performed UHPLC–MS/MS analysis on reactions yielding two or more products.

Overall, when UDP-glucose were used as sugar donors, a greater number of products were generated than using UDP-rhamnose (Fig. 5B). Specifically, when UDP-glucose served as the sugar donor, three diglucosyl and four monoglucosyl products of kaempferol were identified, two main products were identified to kaempferol-3-*O*-glucoside and kaempferol-7-*O*-glucoside when comparing with authentic standards (Fig. 6A, Supplementary Fig. S15). For 8-prenylkaempferol, one monoglucosyl, three diglucosyl, and one triglucosyl products were detected (Fig. 6B, Supplementary Fig. S16). Kaempferide yielded one diglucosyl and two monoglucosyl products (Fig. 6C, Supplementary Fig. S17), while icaritin produced one diglucosyl and one monoglucosyl product (Fig. 6D, Supplementary Fig. S18).

This study further investigated the sugar donor acceptance of UGT72B113 by using different sugar donors. When kaempferol served as the substrate, we tested UDP-glucose, UDP-xylose, UDP-galactose, UDP-rhamnose, and UDP-glucuronic acid as sugar donors. HPLC analysis of the reactions revealed that UGT72B113 could accept UDP-glucose, UDP-xylose, and UDP-galactose as sugar donors, generating one or more products (Fig. 6E). The reaction products of kaempferol and UDP-xylose was also detected by UHPLC–MS/MS. The analysis identified five products, including two dixylosyl and three monoxylosyl derivatives (Supplementary Fig. S19). It can be concluded that UGT72B113 plays a pivotal role in generating the multiple glycosyl types of flavonols.

To further characterize UGT72B113, its subcellular localization was first examined. The UGT72B113-GFP fusion protein co-localized with free mCherry (a marker for the nucleus and cytoplasm), but not with plasma membrane or endoplasmic reticulum markers (Supplementary Fig. S20), indicating that UGT72B113 is a soluble protein predominantly localized in the nucleus and cytosol. Its expression dynamics during leaf development were also analyzed. Transcript levels of *UGT72B113* remained relatively low from stages S1 to S3, followed by an increase at S4 across developmental stages were assessed (Supplementary Fig. S21B). Although all four compounds exhibited similar trends during leaf development, their accumulation did not parallel the expression pattern of *UGT72B113* (Supplementary Fig. S21C and D).

In summary, our results demonstrate that UGT72B113 is a soluble enzyme contributing to flavonol diversity through its catalytic promiscuity. This promiscuity is evident both at the substrate level and in the range of products generated. The enzyme accepts various aglycone substrates, particularly simple kaempferol derivatives, and displays flexibility toward different UDP-sugar donors. In terms of product formation, *in vitro* assays revealed the generation of monoglycosylated products at multiple regio-positions, as well as di-glycosylated or multi-glycosylated products, further expanding the structural diversity of flavonol glycosides in *E. sagittatum*.

## Discussion

### MSI enables higher precision in spatial localization of bioactive compounds in *Epimedium*

Traditionally, determining the optimal medicinal part of a plant involves measuring bioactive compound levels in different tissues. However, MSI now offers a more precise method for directly visualizing the spatial distribution of these compounds, even achieving resolution at the cellular level [21]. Since plant specialized metabolites are often synthesized and stored in specific cell types, integrating MSI with single-cell transcriptomics presents a powerful strategy for elucidating their precise biosynthetic regulation [22].

In *Epimedium*, the aerial parts (leaves and petioles) are used medicinally. Our tissue-specific analysis (Fig. 2A and B) revealed that the marker compounds are significantly more abundant in the leaves that in the petioles, suggesting leaves are the preferable material. However, petioles and leaves are typically harvested together due to the labor involved in separating them in practice. We further investigated whether the distribution within leaves is uniform. MSI revealed heterogeneity, with lower concentrations of epimedin C and icariin in the leaf veins compared to the non-vein part (Fig. 1B). This pattern aligns with the low content found in petioles and suggests that the storage of these two compounds occurs locally within non-vein mesophyll cells. While these findings point to localized storage, whether biosynthesis also occurs exclusively in these cells or involves other tissues cannot be determined from the current data. It is important to acknowledge that MSI provides a static snapshot. The observed distribution could result from a combination of local biosynthesis, differential metabolic turnover, or transport followed by regional sequestration. Future dynamic studies, such as isotope tracing, are needed to disentangle these processes.

Furthermore, cross-sectional MSI of leaves showed a pronounced accumulation of epimedin C and icariin on the spongy tissue, with less on the palisade tissue. This differential distribution may reflect a division of function of adaxial and abaxial cells. Adaxial cells expose to more direct sunlight likely prioritize photosynthesis, while abaxial cells may be more specialized for secondary metabolism. Some studies indicate that the thickness and structure of the palisade tissue on the adaxial side is an important factor affecting the photosynthetic rate of plants. Due to its higher chloroplast content and fewer stomata, the adaxial side serves as the primary site for efficient photosynthesis [23, 24].

While the current MSI setup with 10 μm pixel resolution enables discrimination between major leaf tissue layers (vein and mesophyll, palisade and spongy), it does not provide definitive cellular-level localization. Higher-resolution MSI techniques, such as those coupled with finer laser spot sizes or secondary ion mass spectrometry (SIMS), would be required to resolve metabolite distribution at the single-cell level [21]. Alternatively, complementary approaches such as single-cell metabolomics or fluorescence tagging of biosynthetic enzymes could provide deeper insights into the cell-type-specific accumulation and biosynthesis of these flavonol glycosides in future studies.

### Flavonoid diversity shaped by UGTs exhibits multiple functions and application potential

UGTs are key enzymes driving flavonoid diversity. They catalyze the glycosylation of flavonoid aglycones, transferring sugars such as glucose or rhamnose to modulate solubility, stability, and bioactivity. This modification greatly expands flavonoid structural variety, facilitating ecological adaptation [25]. Functional diversification in this large family arises from gene expansion [11] and neo-functionalization [12]. Notably, UGTs often exhibit substrate promiscuity. A single enzyme can act on multiple flavonoids, and a single flavonoid can be modified by several UGTs [26–28]. This versatility allows plants to dynamically tailor flavonoid profiles in response to developmental and environmental cues, making UGTs central architects of phytochemical diversity.

*In vitro* enzymatic assays demonstrated that UGT72B113 contributes to flavonol diversity through its catalytic promiscuity, accepting diverse acceptor substrates and UDP sugar donors while generating both mono-glycosylated and multi-glycosylated products from a single substrate. However, product identification relied primarily on HPLC and UHPLC–MS/MS with limited authentic standards, allowing determination of glycosyl residue numbers but not the exact regiospecificity of sugar attachment for most products due to the lack of NMR characterization. Additionally, whether multi-glucosylated products arise from stepwise addition or a single transfer event could not be determined from the current data. Although these structural details remain unresolved, the central conclusion that UGT72B113 contributes to flavonol diversity through its broad catalytic promiscuity is well supported, and future mechanistic studies will require more definitive structural characterization. Despite these limitations, this candidate gene remains applicable for future use in synthetic biology or *Epimedium* breeding.

### Functional difference between UGT84AA1 and UGT72B113 arises from structural divergence

In this study, we characterized five UGT candidates, two of them (UGT84AA1 and UGT72B113, Fig. 5B) displayed notable substrate promiscuity. UGT84AA1 catalyzed reactions with all tested substrates, whereas UGT72B113 acted on simpler aglycones (e.g., kaempferol, icaritin) but yielded a greater number and quantity of glycosylated products. We focused on UGT72B113 due to its higher conversion rates and product diversity. Based on our analyses, we propose that UGT72B113 contributes to flavonol diversity in *E. sagittatum* through versatile activity across different substrates, sugar donors, and glycosylation positions. While UGT84AA1 appears to have a narrower functional scope, primarily diversifying substrates, it nonetheless represents another genetic source of flavonol variation.

To explore the structural basis for their functional differences, we performed protein modeling. The active pocket of UGT84AA1 (1616 Å^3^) is larger than that of UGT72B113 (1358 Å^3^), which may explain its ability to accommodate more structurally complex substrates (Supplementary Fig. S22A). Furthermore, the entrance of UGT84AA1 features a flexible loop that likely permits induced-fit deformation for bulky substrates, in contrast to the rigid helical structure at the entrance of UGT72B113. UGT84AA1 also lacks steric gating residues, widening the access path (Supplementary Fig. S22B). In summary, the broader substrate range of UGT84AA1 seems to arise from its larger, more flexible, and more accessible active site. Conversely, the ability of UGT72B113 to generate multiple regio-isomer products from a single substrate may petiole from distinct binding orientations within its more constrained, tunnel-like pocket, possibly aided by a dynamic lid structure. These computational insights suggest that flexibility enhances catalytic adaptability, offering ideas for engineering promiscuous biocatalysts. However, these predictions require further experimental validation in the future.

## Materials and methods

### Materials

*Epimedium sagittatum* plants (grown in South China Botanical Garden, GuangDong, China) were collected and used in this work.

### Sample preparation for MSI

Fresh *E. sagittatum* leaf was embedded in a 10% gelatin (w/v) solution using a silicone mold, the samples were flash-frozen in liquid nitrogen. The frozen blocks were then stored at −80°C for 2 hours and subsequently equilibrated at −20°C for 30 minutes prior to sectioning. Serial sections of 20-μm thickness were consecutively obtained using a Leica CM1950 cryostat (Leica, Germany) at −20°C. The slices were directly placed on conductive indium-tin-oxide (ITO)-coated glass slides and dried in a vacuum desiccator containing desiccant for 2 minutes. Fresh leaves were rinsed with ultrapure water, gently blotted dry to remove residual moisture, and then secured onto conductive ITO-coated glass slides using double-sided adhesive tape. A uniform layer of 1,5-Diaminonaphthalene (1,5-DAN; Sigma, USA) matrix was applied to the sample surface at a thickness of 1.5 μm using a Shimadzu iMLayer matrix vapor deposition system (185°C, 5 minutes; Shimadzu, Japan), followed by a coating with a methanol solution.

### MALDI-TOF MSI analysis

MSI data acquisition was performed on an imaging mass microscope (iMScope QT) system (Shimadzu, Japan) equipped with an optical microscope, an atmospheric pressure MALDI (AP-MALDI) ion source, and a quadrupole time-of-flight (Q-TOF) mass analyzer. The tissue sections were analyzed in positive ion mode with the following MSI parameters: 800 laser shots per pixel at a repetition rate of 5000 Hz; a laser spot diameter of 2 (10 μm); laser intensity, 77.6%; detector voltage, 2.70 kV. The mass spectrometry data were acquired over two consecutive ranges: *m/z* 600 to 900.

### Transcriptome sequencing

Tissue samples were collected from rhizomes, petioles, and leaves, with three biological replicates for each tissue. Subsequent procedures were carried out mainly following in the published paper [29]. RNA extraction, library construction, sequencing, and bioinformatics analysis were performed by Gene Denovo Biotechnology Co., Ltd (Guangzhou, China). Total RNA was extracted using Trizol reagent kit. RNA quality was assessed by Agilent 2100 Bioanalyzer and checked using agarose gel electrophoresis. mRNA was enriched by Oligo(dT) beads, and the enriched mRNA was fragmented into short fragments using fragmentation buffer and reverse transcribed into cDNA with random primers. The cDNA fragments were purified and ligated to Illumina sequencing adapters. Sequencing was performed on Illumina novaseq 6000. The raw data of RNA-seq was submitted to National Genomics Data Center website with accession number CRA037967.

### RNA extraction and gene cloning

For RNA extraction, fresh leaves of *E. sagittatum* were ground into a fine powder under cryogenic conditions using a freezer mill. The resulting powder was then used for total RNA extraction using the HiPure Plant RNA Mini Kit (Magen, China).

The cDNA of *E. sagittatum* leaves was synthesized using StarScript Pro All-in-one RT Mix with gDNA Remover (GenStar, China). Candidate genes were amplified from the cDNA by PCR using PrimeSTAR Max DNA Polymerase (Takara, Japan) and gene-specific primers (Supplementary Table S4). The PCR products were purified by using Siligene Gel DNA Kit (Siligene, China). pET-28a (+) vector (for UGT protein expression) was double-digested with *BamH I* and *Xho I* (New England Biolabs, USA) and purified using the same method. The amplified genes were cloned into the vector by using Fast DNA Assembly Mix (CISTRO, China). After verification of the sequences by Sanger sequencing, the recombinant plasmids were stored at −20°C.

### qRT-PCR analysis

Total RNA of different samples from *E. sagittatum* and reverse-transcribed following the protocols described above. The reaction included former primers, reverse primers, cDNA templates, and the 2 × ChamQ Blue Universal SYBR qPCR Master Mix (Vazyme, China) was performed on a qTower^3^ real-time PCR system (Analytik Jena, Germany). Upon completion of the amplification cycles, a melting curve analysis was conducted to verify the amplification specificity of the target products. Gene-specific primers designed for the qPCR assays are listed in Supplementary Table S4, the *Actin* gene was employed as the reference gene, and relative transcript abundance was determined using the 2^−ΔΔCt^ method [30].

### Recombinant protein expression and purification

The recombinant pET28a-*UGT*s plasmids were transformed into competent cells of *E. coli* BL21(DE3) (Coolaber, China). Then the positive recombinant strains were pre-incubated in 5 ml of Luria-Bertani (LB) media containing 50 μg∙ml^−1^ kanamycin and cultured overnight at 37°C, 2.5 ml overnight cultures were incubated into 250 ml LB media at 37°C and 180 rpm until *OD_600_* reached 0.6–0.8. The recombinant proteins were induced by isopropyl-β-d-thiogalactoside (IPTG). At the same time, auto-induction medium [31] was also used with the same procedure. After 20 hours of incubation at 18°C with constant shaking at 180 rpm, the bacterial cells were collected by centrifugation at 4°C and re-suspended in 40 ml lysis buffer (50 mM Tris–HCl, pH 7.5, containing 0.5 M NaCl, 5 mM imidazole, 5% glycerol, and 1 mM phenylmethylsulfonyl fluoride). After disruption of the cells by sonication on ice (180 W, 5 seconds on/5 seconds off, for a total of 30 min), the lysate was centrifuged at 4°C. The resulting supernatant was filtered through a 0.45-μm membrane and applied to Ni-NTA resin (QIAGEN, Germany) that had been pre-equilibrated with lysis buffer at 4°C. The recombinant proteins were then purified with wash buffer (50 mM Tris–HCl, pH 7.5, containing 0.5 M NaCl, 50 mM imidazole, 5% glycerol), and the target proteins were eluted with elution buffer (50 mM Tris–HCl, pH 7.5, containing 0.5 M NaCl, 50 mM imidazole, 5% glycerol). The target protein was concentrated and buffer was exchanged into desalting buffer (50 mM Tris–HCl, pH 7.5, containing 1 mM dithiothreitol, 10% glycerol) using an Amicon Ultra-30 kDa centrifugal filter (Merck Millipore, USA). The purified proteins were analyzed by SDS-PAGE, and protein concentration was determined by the Bradford Protein Assay Kit (Beyotime, China).

### Enzyme activity assay

For crude enzyme: Induced bacterial cells were harvested by centrifugation (12 000 rpm, 4°C), resuspended in 50 mM Tris–HCl (pH 7.5), and lysed by sonication. The crude enzyme extract was obtained as the supernatant after centrifugation (12 000 rpm, 20 min, 4°C) of the lysate. The 100-μl reaction mixture, containing the crude enzyme, 1 mM sugar donor (UDP-glucose and UDP-rhamnose, Yuanye, China), and 0.5 mM acceptor substrate, was incubated at 35°C for 3 hours. After quenching the reaction with 200 μl of methanol, the mixture was centrifuged (12 000 rpm, 15 minutes), followed by filtration through a 0.22-μm membrane and subsequent HPLC analysis.

For purified enzyme: The *in vitro* assays for UGTs were performed in a 50-μl reaction volume containing 50 mM Tris–HCl (pH 7.5), 1 mM sugar donor (UDP-glucose, UDP-rhamnose, UDP-xylose, UDP-galactose, or UDP- glucuronic acid; all from Yuanye, China), 0.5 mM acceptor substrate, and 10 μg of purified recombinant protein. The reaction mixture was gently mixed and incubated at 35°C for 3 hours. The reaction was terminated by adding 100 μl of methanol. The resulting solution was centrifuged at 12 000 rpm for 10 minutes, passed through a 0.22-μm membrane filter, and subsequently analyzed by HPLC and LC–MS. A negative control was performed under identical conditions using heat-inactivated enzyme.

The acceptor substrates include kaempferol, kaempferide, 8-prenylkaempferol, icaritin, icariside I, icariside II, and icariin. All these compounds were purchased from Chengdu Desite Biological Technology Co., Ltd, China.

For kinetic assays of UGT72B113: It was performed in 100-μl reactions (50 mM Tris–HCl, pH 7.5; 2 mM UDP-Glc; 5–80 μM substrate) at 35°C. Enzyme loadings and incubation times were tailored per substrate: 200 ng for 2 to 5 minutes (kaempferol and kaempferide) and 1 μg for 10 minutes (8-prenylkaempferol). All reactions were quenched with two volumes of ice-cold methanol. Due to the unavailability of commercial standards for the glycosylated products, their concentrations were quantified based on the standard curves of the respective substrates, assuming equivalent molar response factors. All data are shown as the mean ± standard deviation (SD) from three independent replicates. Kinetic constants (*K*_m_ and *V*_max_) were determined by fitting the initial reaction velocities (*v*) to the Michaelis–Menten equation via non-linear regression:


$$ v=\frac{V_{\mathrm{m}\mathrm{ax}}\cdotp \left[S\right]}{K_{\mathrm{m}}+\left[S\right]} $$

where [*S*] is the substrate concentration.

### HPLC and UPLC-MS analysis

HPLC analysis of the reaction products was conducted on a Shimadzu LC-2030C 3D system (Shimadzu, Japan) using an InertSustain AQ-C18 column (4.6 × 150 mm, 5 μm). The methods employed a flow of 1 ml/min, with the column temperature set to 40°C and an injection volume of 10 μl. The gradient elution mobile phase consisted of H_2_O with 0.01% acetic acid (Solvent A) and acetonitrile (Solvent B). The elution ran in the following conditions: 0–3 minutes, 20% B; 3–16 minutes, 20%–50% B; 16–18 minutes, 50% B; 18–28 minutes, 50%–80% B; 28–29 minutes, 80%–95% B; 29–33 minutes, 95%, B; 33–34 minutes 95%–20% B.

UPLC-MS analysis was performed on a Thermo Scientific Orbitrap Elite UPLC-MS–MS system (Thermo Scientific, U.S.A.) equipped with a UPLC Hypersil Gold column (100 × 2.1 mm, 1.9 μm). The analysis was carried out at 40°C with a flow rate of 0.3 ml/min and an injection volume of 1 μl. The gradient elution conditions were the same as those used for HPLC. The mass spectrometer operation parameters were set as follows: ion source temperature 300°C, sheath gas 35 arb, aux gas 10 arb, ion transfer tube temperature 275°C, ion spray voltage 2.5 KV for negative mode, and 3.5 KV for positive mode, and mass scan range (*m/z*) of 200 to 900.

### Subcellular localization of the UGT72B113 protein

The full-length coding sequence of UGT72B113 was cloned into the pSuper1300-GFP vector to generate a C-terminal GFP-fusion construct. The recombinant plasmid was transformed into *Agrobacterium tumefaciens* strain GV3101 and subsequently co-infiltrated with various organelle markers genes into the leaves of 4-week-old *Nicotiana benthamiana*. Fluorescent signals were captured 48 to 72 hours post-infiltration using a Leica SP8 STED 3× laser confocal microscope (Leica Microsystems, Germany). GFP and mCherry were excited at 488 and 561 nm, with emission signals detected at 500 to 530 nm and 580 to 630 nm, respectively.

### Data analysis

Data analysis was performed using the proprietary software. Optical and mass spectrometry imaging data were analyzed with IMAGEREVEAL MS (Shimadzu, Japan). HPLC data were processed using the LabSolution software (Shimadzu, Japan) and UPLC-MS data were analyzed with Xcalibur Qual Browser (Thermo Fisher Scientific, USA). The peak normalization and peak area calculation was performed by software with default setting. Statistical significance was determined by Student's *t*-test using Microsoft Excel and was visualized using DMSAS software (version 1.10.0).

### Protein structure prediction and comparative analysis

The tertiary structures of UGT72B113 and UGT84AA1 were predicted using AlphaFold 3. Model reliability was assessed by calculating global average pLDDT scores derived from the B-factor fields of the generated .cif files, with per-residue confidence profiles visualized using Python. Substrate-binding pockets were characterized using the CASTpFold server, where the primary active site was identified based on the largest solvent-accessible volume and spatial proximity to conserved catalytic residues. Structural alignment and visualization were performed using PyMOL (Incentive Product, Version 3.1.3.1, Schrödinger, LLC). Following global superposition based on conserved Cα atoms, the geometric dimensions of the pocket entrance were analyzed for steric bottlenecks. Furthermore, structural divergences in the variable N-terminal and loop regions were characterized and cross-referenced with local pLDDT scores to evaluate the correlation between structural conformation and intrinsic flexibility.

## Supplementary Material

Web_Material_uhag187

## Data Availability

The raw data of RNA-seq was submitted to National Genomics Data Center website with accession number CRA037967. Other data underlying this article will be shared on reasonable requests to the corresponding author.
