# Supplementary material for: Spatial distribution of marker compounds in Epimedium sagittatum leaves and the role of UGT72B113 in generating kaempferol glycoside diversity
Source: Hortic Res. 2026 May 9;13(10):uhag187. doi: 10.1093/hr/uhag187 (PMC13610573; doi:10.1093/hr/uhag187)
Supplement: Supplementary file 1 [file web_material_uhag187.zip › FigS1.pdf]

A

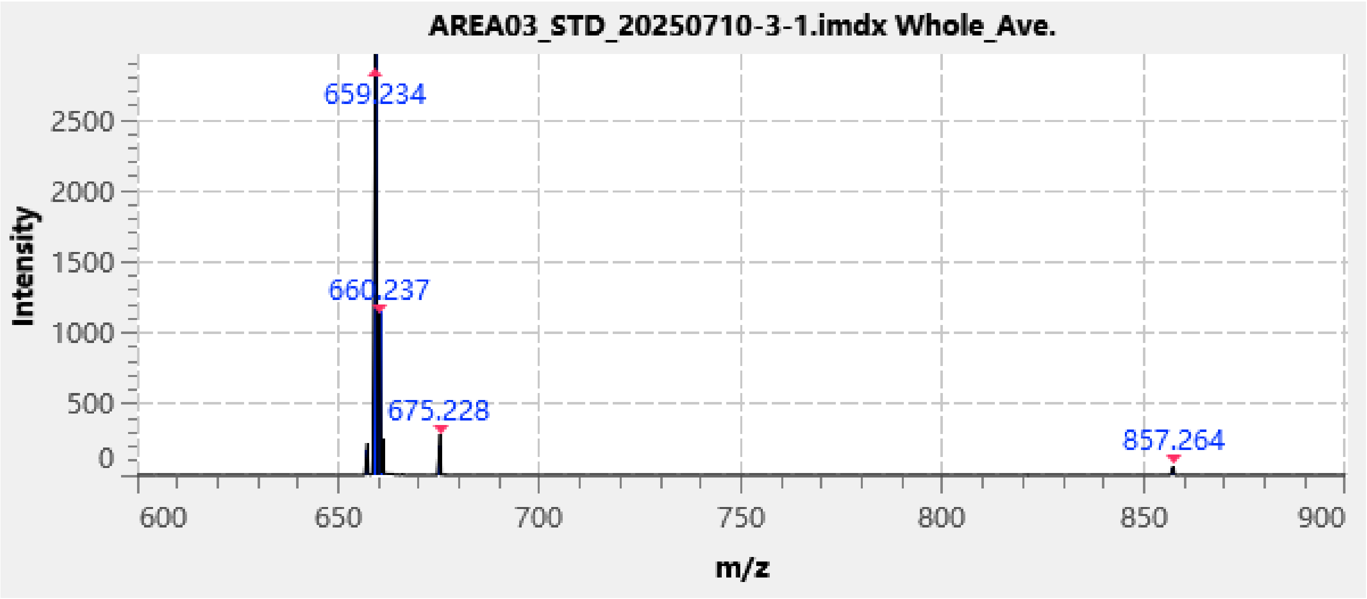

AREA03_STD_20250710-3-1.imdx Whole_Ave.
Intensity
2500
2000
1500
1000
500
0
659.234
660.237
675.228
857.264
600
650
700
750
800
850
900
m/z

B

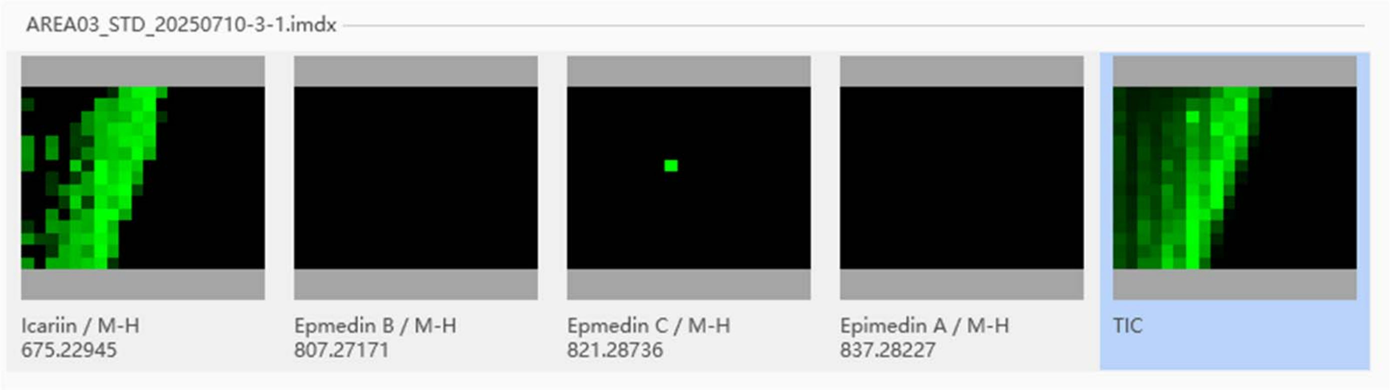

AREA03_STD_20250710-3-1.imdx
Icariin / M-H
675.22945
Epmedin B / M-H
807.27171
Epmedin C / M-H
821.28736
Epimedin A / M-H
837.28227
TIC
