# Supplementary material for: Spatial distribution of marker compounds in Epimedium sagittatum leaves and the role of UGT72B113 in generating kaempferol glycoside diversity
Source: Hortic Res. 2026 May 9;13(10):uhag187. doi: 10.1093/hr/uhag187 (PMC13610573; doi:10.1093/hr/uhag187)
Supplement: Supplementary file 1 [file web_material_uhag187.zip › FigS4.pdf]

A

B

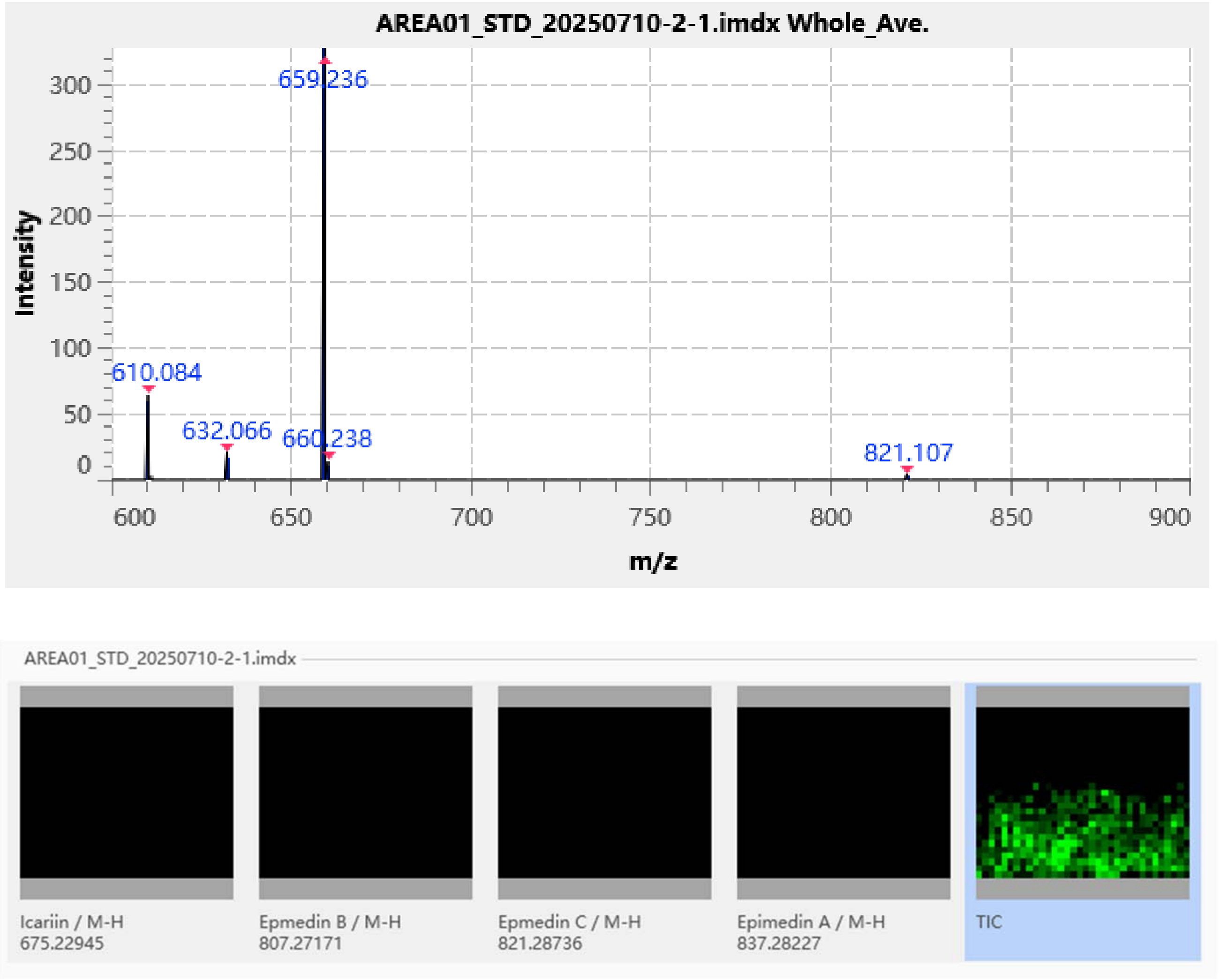
AREA01_STD_20250710-2-1.imdx Whole_Ave.
Intensity
m/z
659.236
610.084
632.066
660.238
821.107
AREA01_STD_20250710-2-1.imdx
Icariin / M-H
675.22945
Epmedin B / M-H
807.27171
Epmedin C / M-H
821.28736
Epimedin A / M-H
837.28227
TIC
