# Supplementary material for: Spatial distribution of marker compounds in Epimedium sagittatum leaves and the role of UGT72B113 in generating kaempferol glycoside diversity
Source: Hortic Res. 2026 May 9;13(10):uhag187. doi: 10.1093/hr/uhag187 (PMC13610573; doi:10.1093/hr/uhag187)
Supplement: Supplementary file 1 [file web_material_uhag187.zip › FigS7.pdf]

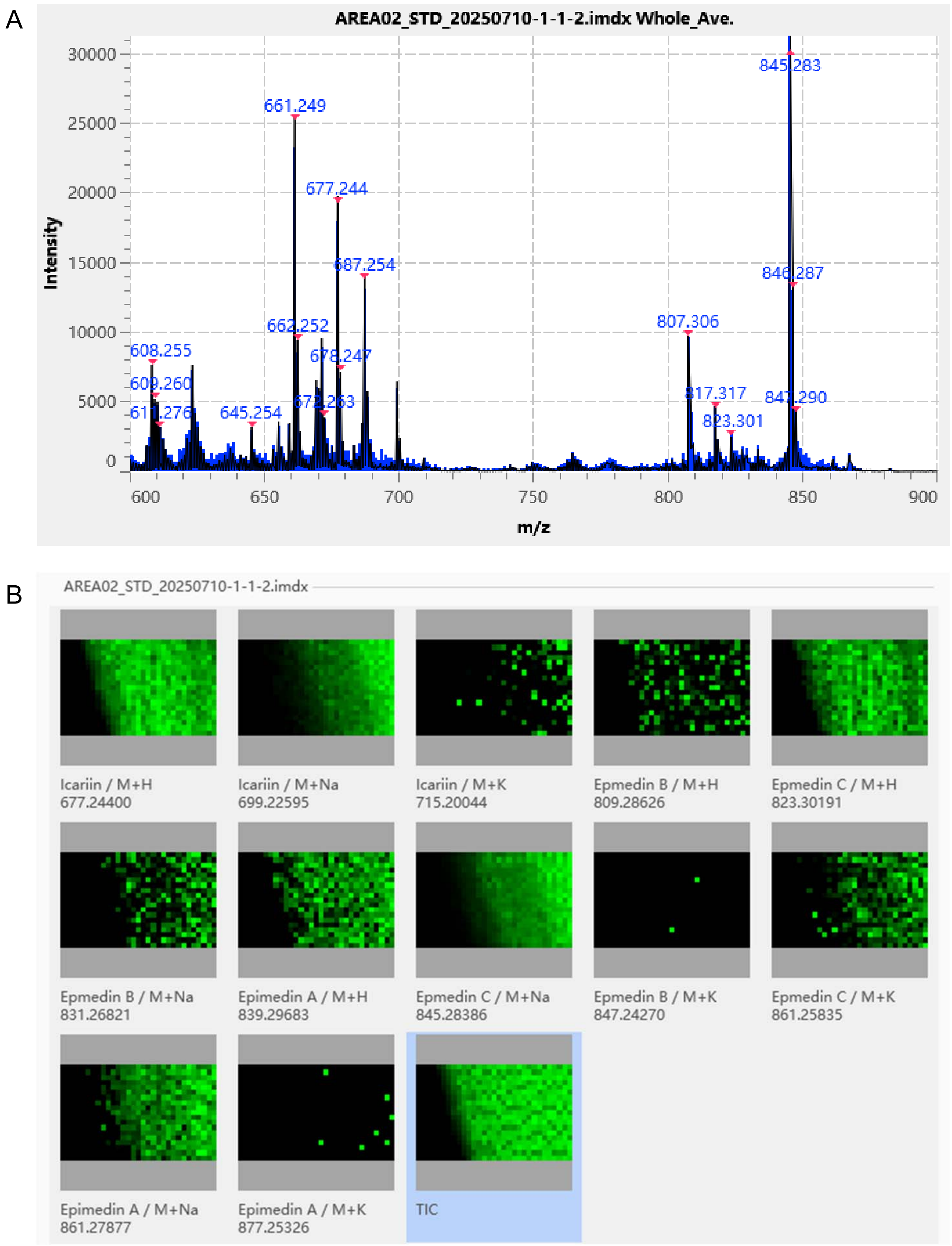
A
AREA02_STD_20250710-1-1-2.imdx Whole_Ave.
Intensity
m/z
845.283
661.249
677.244
687.254
846.287
807.306
662.252
608.255
678.247
609.260
817.317
847.290
672.263
611.276
645.254
823.301
B
AREA02_STD_20250710-1-1-2.imdx
Icariin / M+H
677.24400
Icariin / M+Na
699.22595
Icariin / M+K
715.20044
Epmedin B / M+H
809.28626
Epmedin C / M+H
823.30191
Epmedin B / M+Na
831.26821
Epmedin A / M+H
839.29683
Epmedin C / M+Na
845.28386
Epmedin B / M+K
847.24270
Epmedin C / M+K
861.25835
Epimedin A / M+Na
861.27877
Epimedin A / M+K
877.25326
TIC
