# Supplementary material for: Spatial distribution of marker compounds in Epimedium sagittatum leaves and the role of UGT72B113 in generating kaempferol glycoside diversity
Source: Hortic Res. 2026 May 9;13(10):uhag187. doi: 10.1093/hr/uhag187 (PMC13610573; doi:10.1093/hr/uhag187)
Supplement: Supplementary file 1 [file web_material_uhag187.zip › FigS8.pdf]

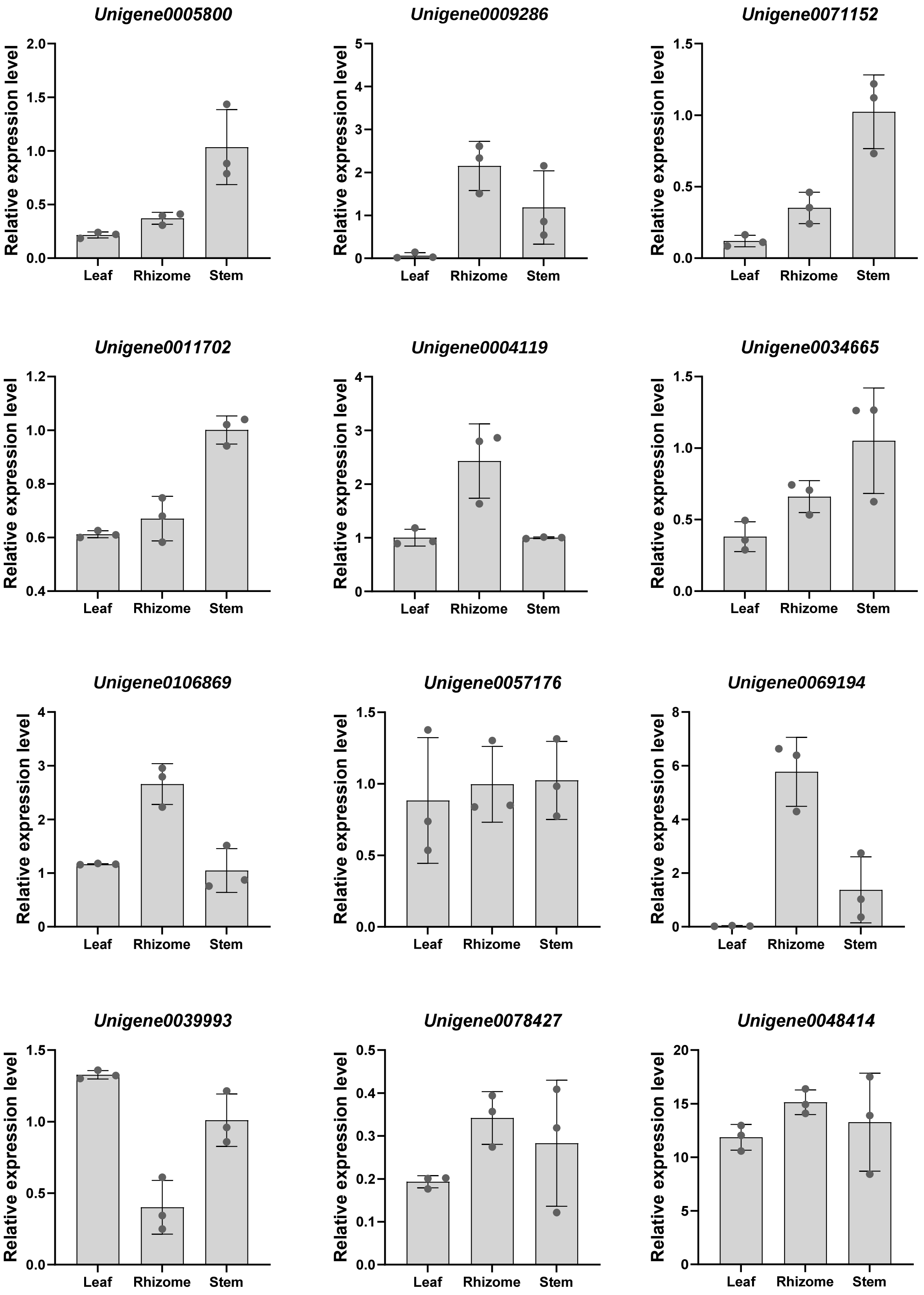
Unigene0005800
Unigene0009286
Unigene0071152
Unigene0011702
Unigene0004119
Unigene0034665
Unigene0106869
Unigene0057176
Unigene0069194
Unigene0039993
Unigene0078427
Unigene0048414
Relative expression level
Leaf
Rhizome
Stem
