# Supplementary material for: Spatial distribution of marker compounds in Epimedium sagittatum leaves and the role of UGT72B113 in generating kaempferol glycoside diversity
Source: Hortic Res. 2026 May 9;13(10):uhag187. doi: 10.1093/hr/uhag187 (PMC13610573; doi:10.1093/hr/uhag187)
Supplement: Supplementary file 1 [file web_material_uhag187.zip › FigS9.pdf]

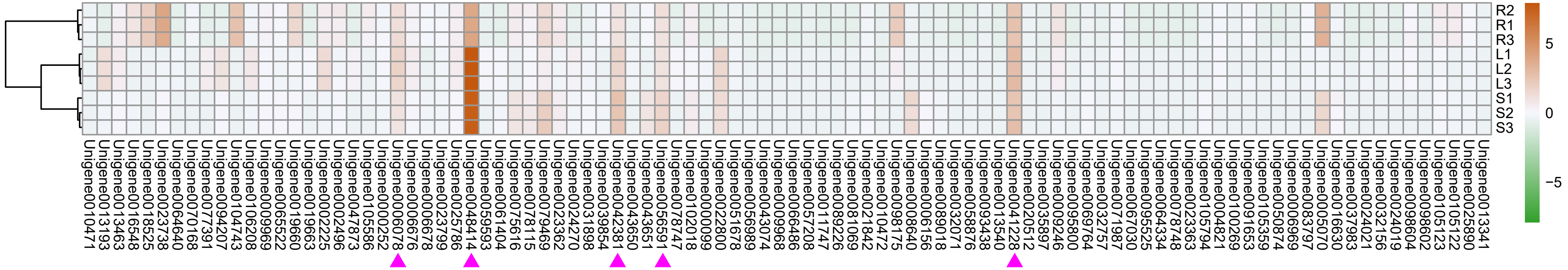
R2
R1
R3
L1
L2
L3
S1
S2
S3
5
0
−5
Unigene0010471
Unigene0013193
Unigene0013463
Unigene0016548
Unigene0018525
Unigene0023738
Unigene0064640
Unigene0070168
Unigene0077391
Unigene0094207
Unigene0104743
Unigene0106208
Unigene0009969
Unigene0065522
Unigene0019660
Unigene0019663
Unigene0002225
Unigene0002496
Unigene0047873
Unigene0105586
Unigene0000252
Unigene0006078
Unigene0006676
Unigene0006678
Unigene0023799
Unigene0025786
Unigene0048414
Unigene0059593
Unigene0061404
Unigene0075616
Unigene0078115
Unigene0079469
Unigene0023362
Unigene0024270
Unigene0031898
Unigene0039854
Unigene0042381
Unigene0043650
Unigene0043651
Unigene0056591
Unigene0078747
Unigene0102018
Unigene0000099
Unigene0022800
Unigene0051678
Unigene0056989
Unigene0043074
Unigene0009968
Unigene0066486
Unigene0057208
Unigene0011747
Unigene0089226
Unigene0081069
Unigene0021842
Unigene0010472
Unigene0098175
Unigene0008640
Unigene0006156
Unigene0089018
Unigene0032071
Unigene0058876
Unigene0093438
Unigene0013540
Unigene0041228
Unigene0020512
Unigene0035897
Unigene0009246
Unigene0095800
Unigene0069764
Unigene0032757
Unigene0071987
Unigene0067030
Unigene0095525
Unigene0064334
Unigene0078748
Unigene0023363
Unigene0105794
Unigene0004821
Unigene0100269
Unigene0091653
Unigene0105359
Unigene0050874
Unigene0006969
Unigene0083797
Unigene0005070
Unigene0016630
Unigene0037983
Unigene0032156
Unigene0024021
Unigene0024019
Unigene0098602
Unigene0105123
Unigene0105122
Unigene0025890
Unigene0013341
