# Supplementary material for: Spatial distribution of marker compounds in Epimedium sagittatum leaves and the role of UGT72B113 in generating kaempferol glycoside diversity
Source: Hortic Res. 2026 May 9;13(10):uhag187. doi: 10.1093/hr/uhag187 (PMC13610573; doi:10.1093/hr/uhag187)
Supplement: Supplementary file 1 [file web_material_uhag187.zip › FigS10.pdf]

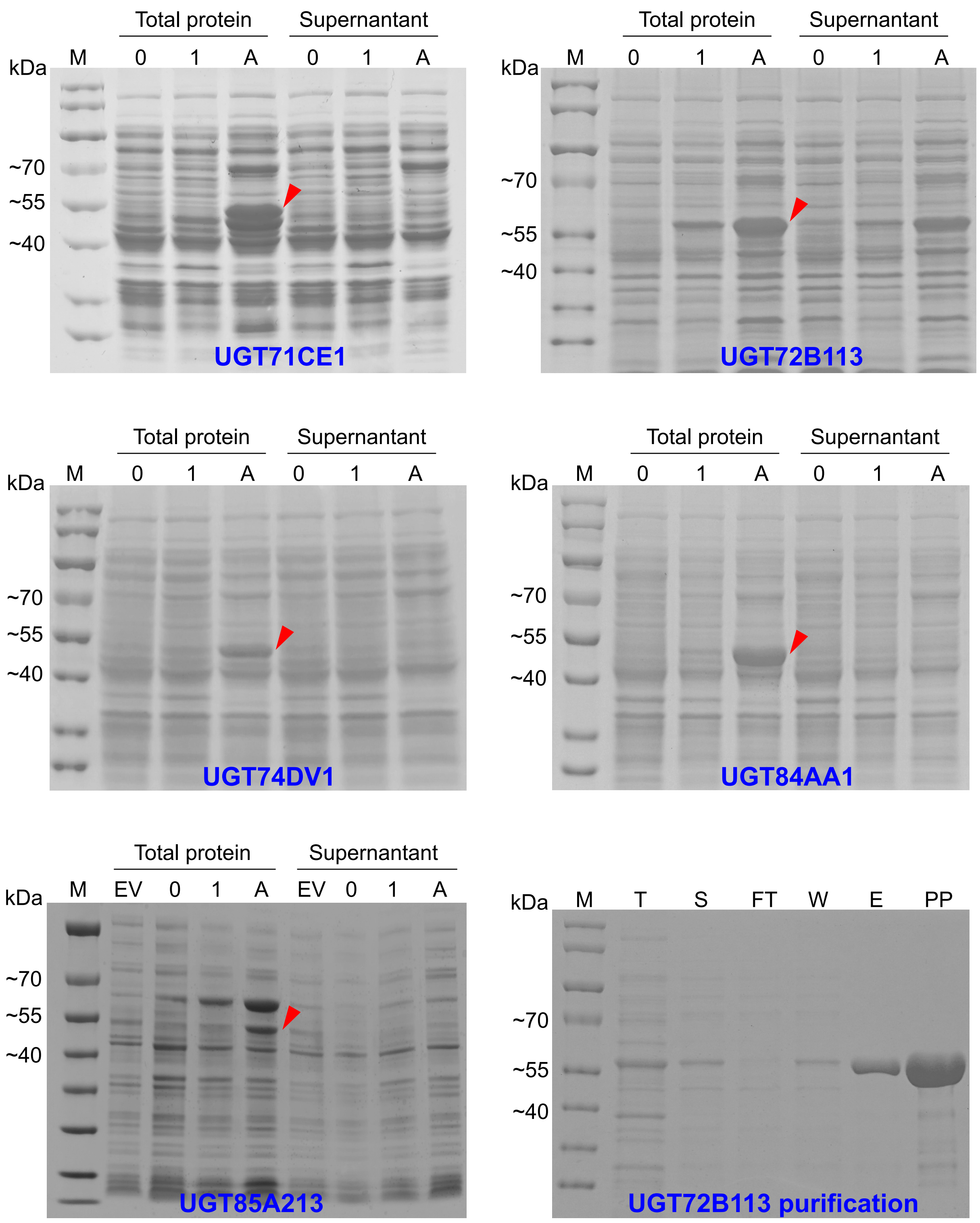
Total protein
Supernatant
kDa
M
0
1
A
0
1
A
~70
~55
~40
UGT71CE1
Total protein
Supernatant
kDa
M
0
1
A
0
1
A
~70
~55
~40
UGT72B113
Total protein
Supernatant
kDa
M
0
1
A
0
1
A
~70
~55
~40
UGT74DV1
Total protein
Supernatant
kDa
M
0
1
A
0
1
A
~70
~55
~40
UGT84AA1
Total protein
Supernatant
kDa
M
EV
0
1
A
EV
0
1
A
~70
~55
~40
UGT85A213
kDa
M
T
S
FT
W
E
PP
~70
~55
~40
UGT72B113 purification
