# Supplementary material for: Spatial distribution of marker compounds in Epimedium sagittatum leaves and the role of UGT72B113 in generating kaempferol glycoside diversity
Source: Hortic Res. 2026 May 9;13(10):uhag187. doi: 10.1093/hr/uhag187 (PMC13610573; doi:10.1093/hr/uhag187)
Supplement: Supplementary file 1 [file web_material_uhag187.zip › FigS12.pdf]

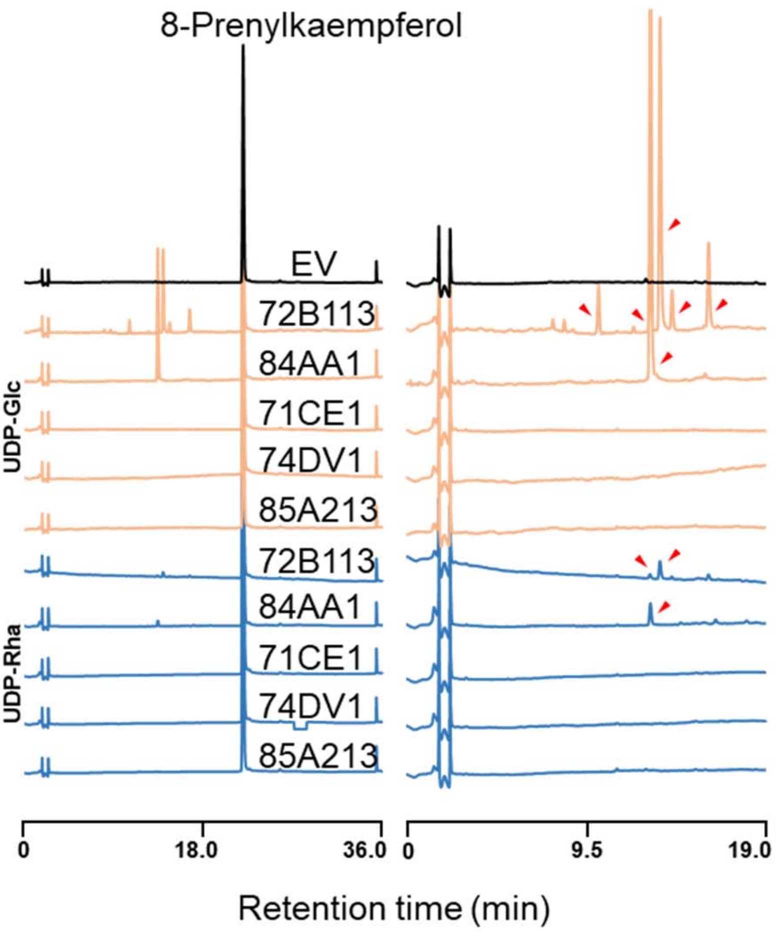

8-Prenylkaempferol
EV
72B113
84AA1
71CE1
74DV1
85A213
72B113
84AA1
71CE1
74DV1
85A213
UDP-Glc
UDP-Rha
0
18.0
36.0
0
9.5
19.0
Retention time (min)


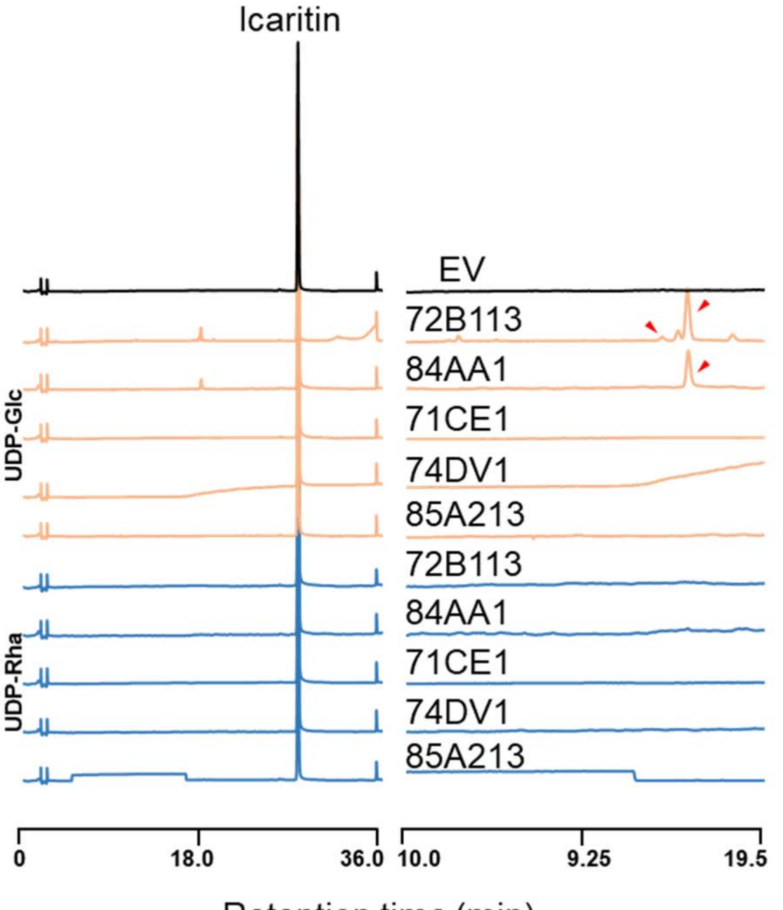

Icaritin
EV
72B113
84AA1
71CE1
74DV1
85A213
72B113
84AA1
71CE1
74DV1
85A213
UDP-Glc
UDP-Rha
0
18.0
36.0
10.0
9.25
19.5
Retention time (min)
