# Supplementary material for: Spatial distribution of marker compounds in Epimedium sagittatum leaves and the role of UGT72B113 in generating kaempferol glycoside diversity
Source: Hortic Res. 2026 May 9;13(10):uhag187. doi: 10.1093/hr/uhag187 (PMC13610573; doi:10.1093/hr/uhag187)
Supplement: Supplementary file 1 [file web_material_uhag187.zip › FigS14.pdf]

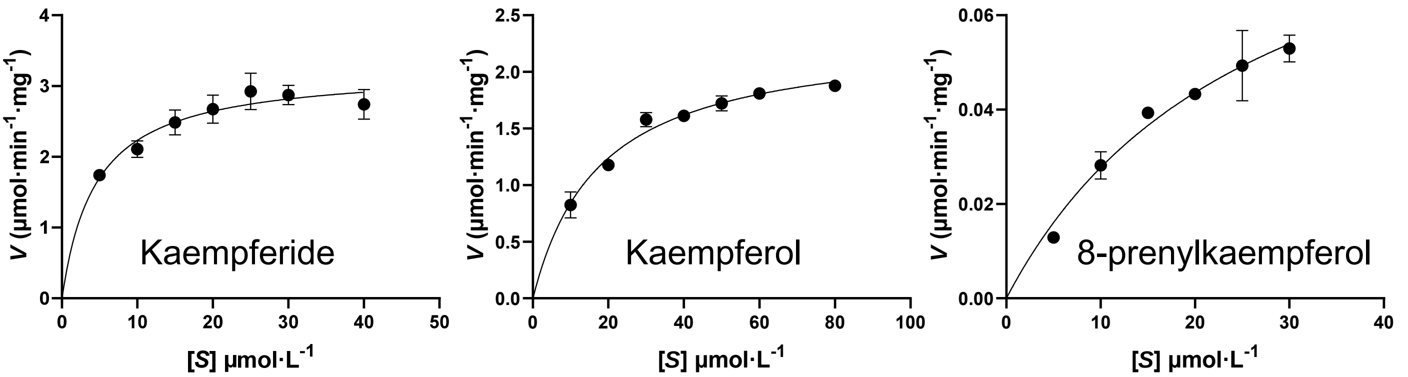

Substrate	K_m (μmol·L^{-1})	V_{max} (μmol·min^{-1}·mg^{-1})	K_{cat} (s^{-1})	k_{cat}/K_m (M^{-1}·s^{-1})
Kaempferide	4.51 ± 0.76	3.24 ± 0.12	2.79	6.19×10^5
Kaempferol	17.64 ± 1.63	2.33 ± 0.068	2.01	1.14×10^5
8-prenylkaempferol	26.80 ± 6.26	0.10 ± 0.013	0.09	3.36×10^3
Icaritin	n.d.	n.d.	n.d.	n.d.
