# Supplementary material for: Spatial distribution of marker compounds in Epimedium sagittatum leaves and the role of UGT72B113 in generating kaempferol glycoside diversity
Source: Hortic Res. 2026 May 9;13(10):uhag187. doi: 10.1093/hr/uhag187 (PMC13610573; doi:10.1093/hr/uhag187)
Supplement: Supplementary file 1 [file web_material_uhag187.zip › FigS15.pdf]

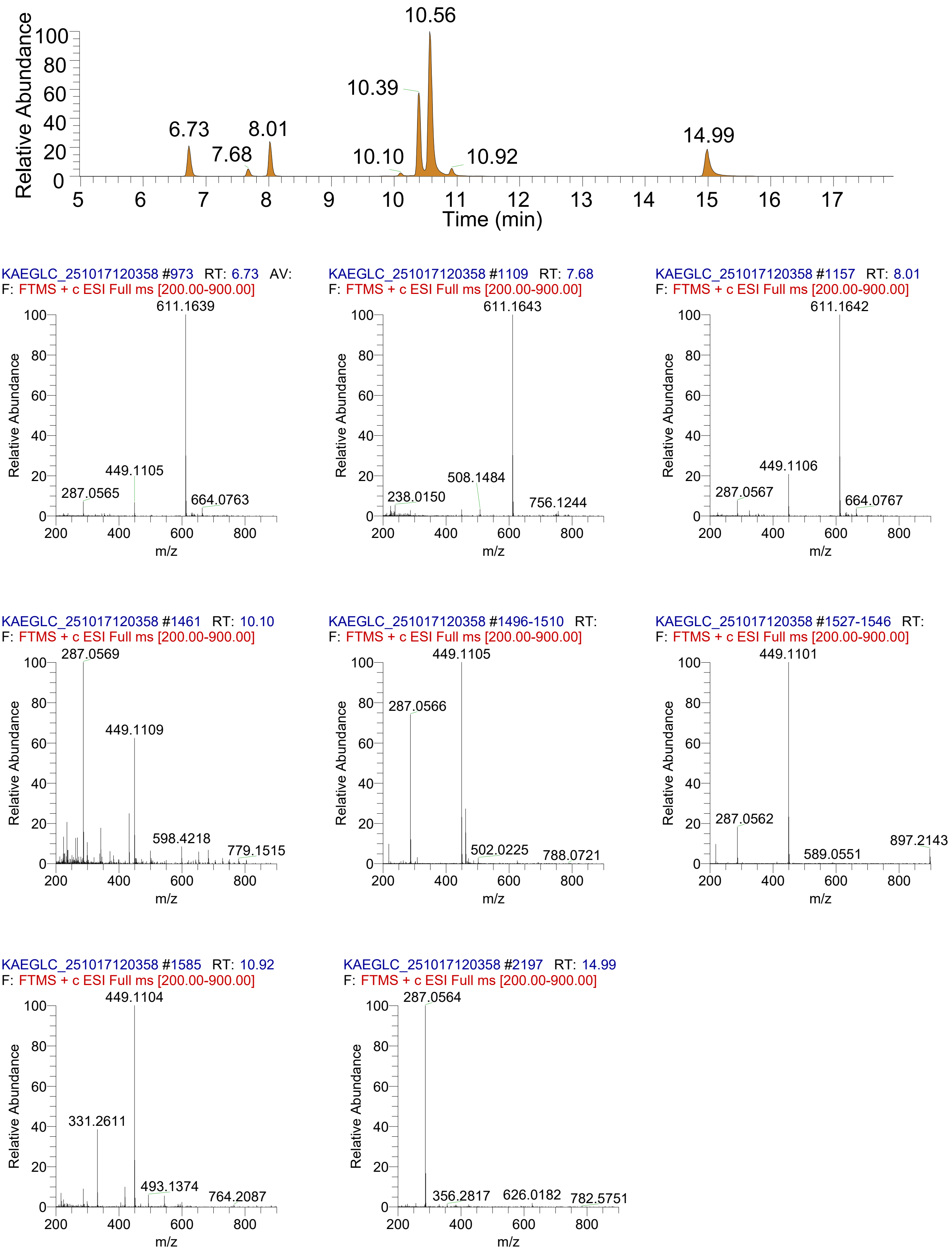

Relative Abundance
Time (min)
6.73
7.68
8.01
10.10
10.39
10.56
10.92
14.99
KAEGLC_251017120358 #973 RT: 6.73 AV:
F: FTMS + c ESI Full ms [200.00-900.00]
611.1639
449.1105
287.0565
664.0763
KAEGLC_251017120358 #1109 RT: 7.68
F: FTMS + c ESI Full ms [200.00-900.00]
611.1643
508.1484
238.0150
756.1244
KAEGLC_251017120358 #1157 RT: 8.01
F: FTMS + c ESI Full ms [200.00-900.00]
611.1642
449.1106
287.0567
664.0767
KAEGLC_251017120358 #1461 RT: 10.10
F: FTMS + c ESI Full ms [200.00-900.00]
287.0569
449.1109
598.4218
779.1515
KAEGLC_251017120358 #1496-1510 RT:
F: FTMS + c ESI Full ms [200.00-900.00]
449.1105
287.0566
502.0225
788.0721
KAEGLC_251017120358 #1527-1546 RT:
F: FTMS + c ESI Full ms [200.00-900.00]
449.1101
287.0562
589.0551
897.2143
KAEGLC_251017120358 #1585 RT: 10.92
F: FTMS + c ESI Full ms [200.00-900.00]
449.1104
331.2611
493.1374
764.2087
KAEGLC_251017120358 #2197 RT: 14.99
F: FTMS + c ESI Full ms [200.00-900.00]
287.0564
356.2817
626.0182
782.5751
m/z
