# Supplementary material for: Spatial distribution of marker compounds in Epimedium sagittatum leaves and the role of UGT72B113 in generating kaempferol glycoside diversity
Source: Hortic Res. 2026 May 9;13(10):uhag187. doi: 10.1093/hr/uhag187 (PMC13610573; doi:10.1093/hr/uhag187)
Supplement: Supplementary file 1 [file web_material_uhag187.zip › FigS16.pdf]

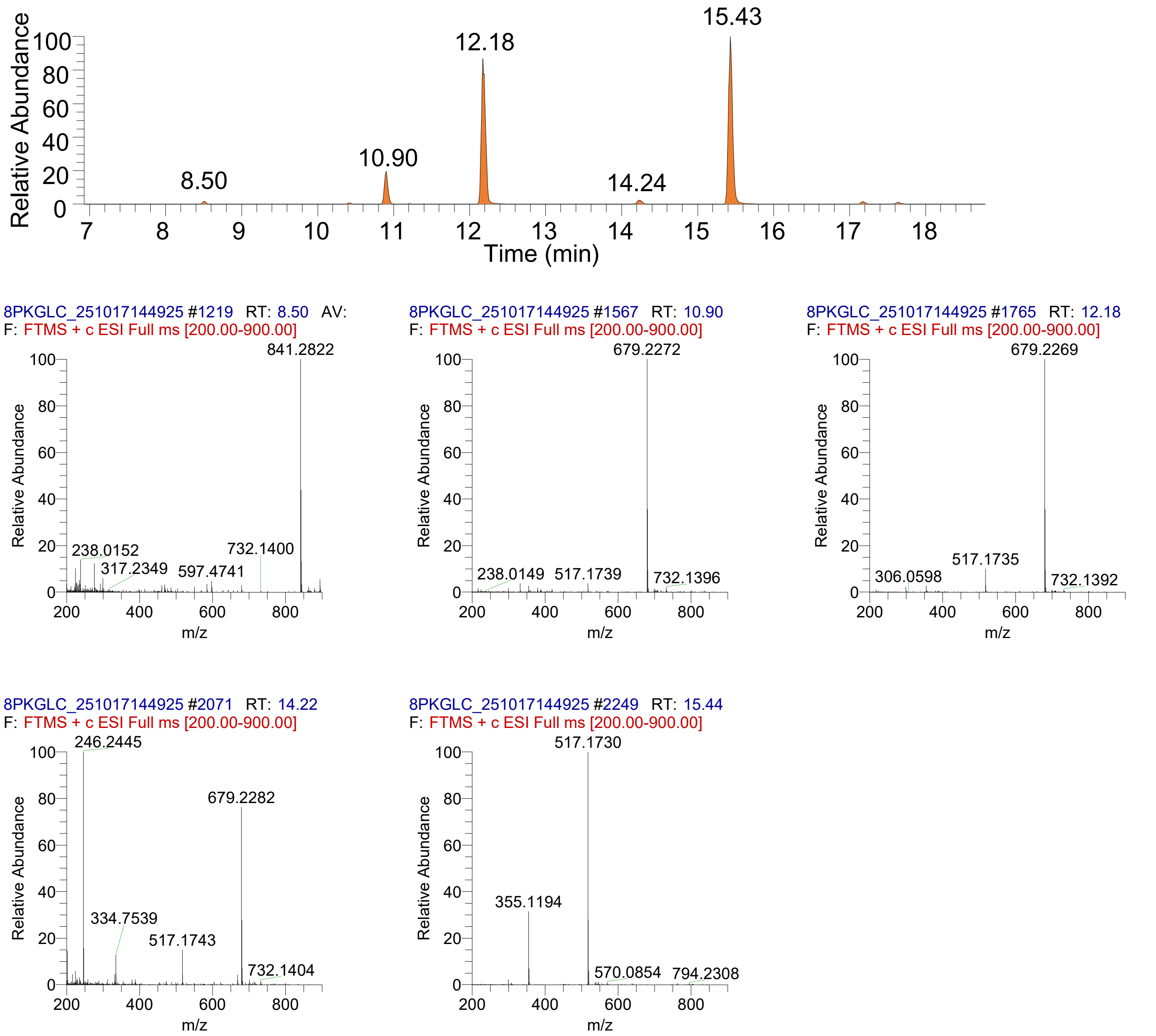

Relative Abundance
Time (min)
8.50
10.90
12.18
14.24
15.43
8PKGLC_251017144925 #1219 RT: 8.50 AV:
F: FTMS + c ESI Full ms [200.00-900.00]
841.2822
238.0152
317.2349
597.4741
732.1400
8PKGLC_251017144925 #1567 RT: 10.90
F: FTMS + c ESI Full ms [200.00-900.00]
679.2272
238.0149
517.1739
732.1396
8PKGLC_251017144925 #1765 RT: 12.18
F: FTMS + c ESI Full ms [200.00-900.00]
679.2269
306.0598
517.1735
732.1392
8PKGLC_251017144925 #2071 RT: 14.22
F: FTMS + c ESI Full ms [200.00-900.00]
246.2445
679.2282
334.7539
517.1743
732.1404
8PKGLC_251017144925 #2249 RT: 15.44
F: FTMS + c ESI Full ms [200.00-900.00]
517.1730
355.1194
570.0854
794.2308
m/z
