# Supplementary material for: Spatial distribution of marker compounds in Epimedium sagittatum leaves and the role of UGT72B113 in generating kaempferol glycoside diversity
Source: Hortic Res. 2026 May 9;13(10):uhag187. doi: 10.1093/hr/uhag187 (PMC13610573; doi:10.1093/hr/uhag187)
Supplement: Supplementary file 1 [file web_material_uhag187.zip › FigS17.pdf]

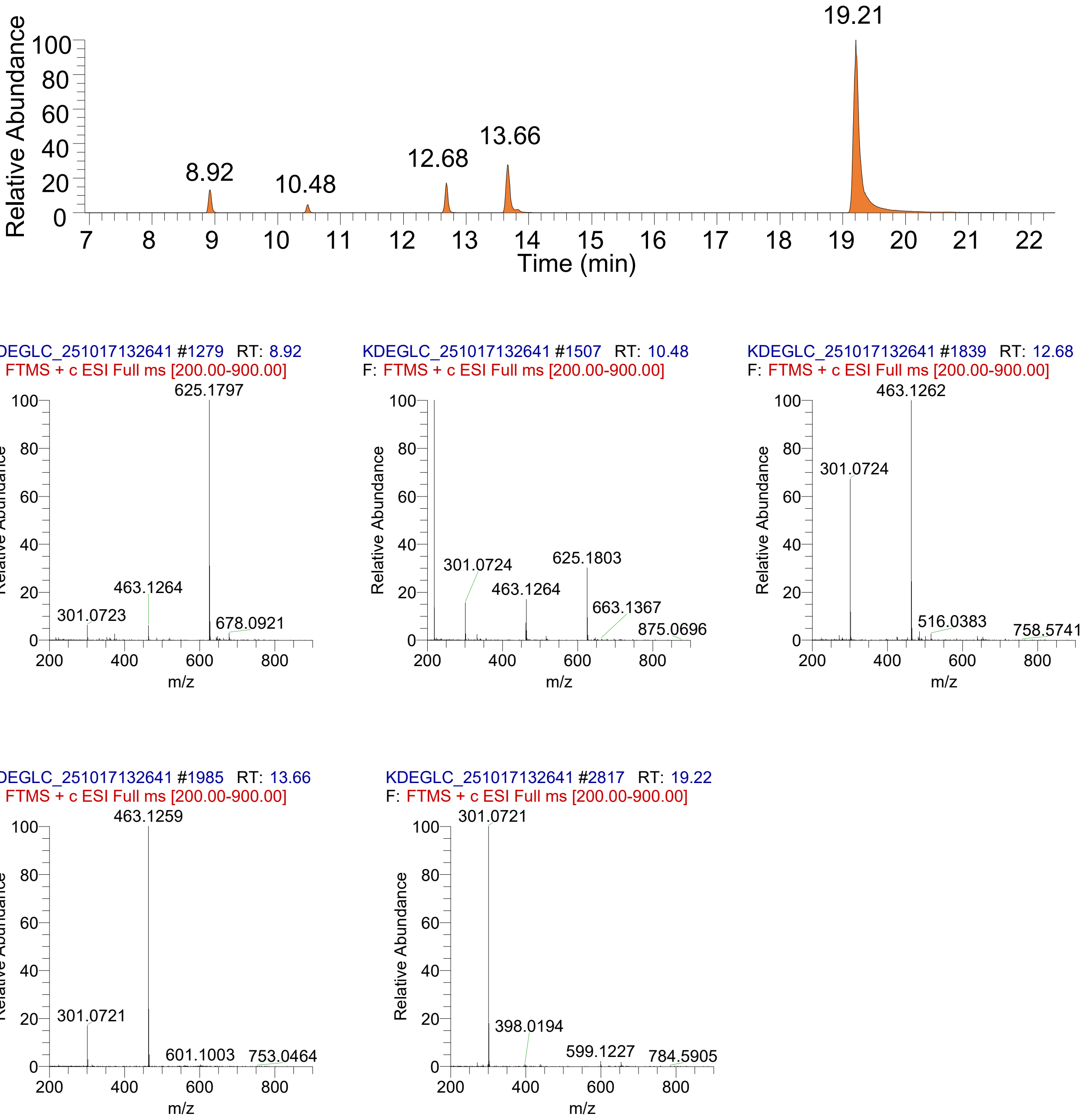
Relative Abundance
Time (min)
19.21
13.66
12.68
10.48
8.92
KDEGLC_251017132641 #1279 RT: 8.92
F: FTMS + c ESI Full ms [200.00-900.00]
625.1797
463.1264
301.0723
678.0921
KDEGLC_251017132641 #1507 RT: 10.48
F: FTMS + c ESI Full ms [200.00-900.00]
301.0724
625.1803
463.1264
663.1367
875.0696
KDEGLC_251017132641 #1839 RT: 12.68
F: FTMS + c ESI Full ms [200.00-900.00]
463.1262
301.0724
516.0383
758.5741
KDEGLC_251017132641 #1985 RT: 13.66
F: FTMS + c ESI Full ms [200.00-900.00]
463.1259
301.0721
601.1003
753.0464
KDEGLC_251017132641 #2817 RT: 19.22
F: FTMS + c ESI Full ms [200.00-900.00]
301.0721
398.0194
599.1227
784.5905
m/z
