# Supplementary material for: Spatial distribution of marker compounds in Epimedium sagittatum leaves and the role of UGT72B113 in generating kaempferol glycoside diversity
Source: Hortic Res. 2026 May 9;13(10):uhag187. doi: 10.1093/hr/uhag187 (PMC13610573; doi:10.1093/hr/uhag187)
Supplement: Supplementary file 1 [file web_material_uhag187.zip › FigS18.pdf]

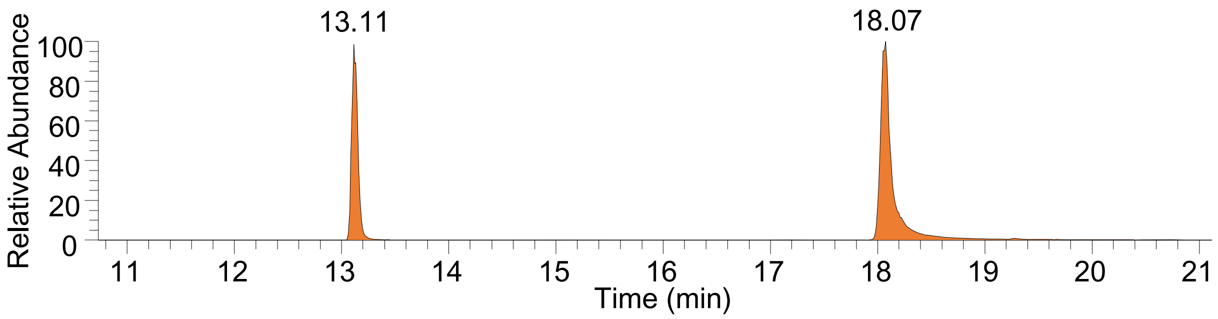
13.11
18.07
Relative Abundance
100
80
60
40
20
0
11
12
13
14
15
16
17
18
19
20
21
Time (min)

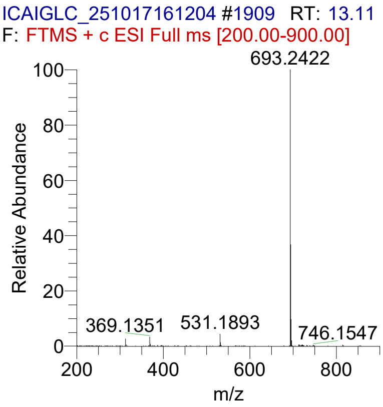
ICAIGLC_251017161204 #1909 RT: 13.11
F: FTMS + c ESI Full ms [200.00-900.00]
693.2422
Relative Abundance
100
80
60
40
20
0
369.1351
531.1893
746.1547
200
400
600
800
m/z

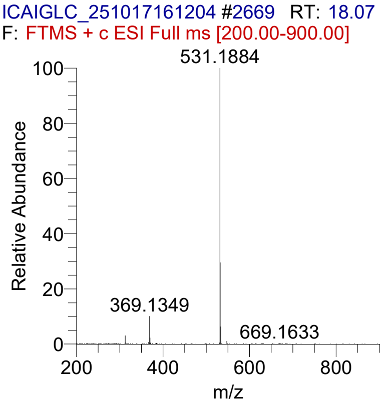
ICAIGLC_251017161204 #2669 RT: 18.07
F: FTMS + c ESI Full ms [200.00-900.00]
531.1884
Relative Abundance
100
80
60
40
20
0
369.1349
669.1633
200
400
600
800
m/z
