# Supplementary material for: Spatial distribution of marker compounds in Epimedium sagittatum leaves and the role of UGT72B113 in generating kaempferol glycoside diversity
Source: Hortic Res. 2026 May 9;13(10):uhag187. doi: 10.1093/hr/uhag187 (PMC13610573; doi:10.1093/hr/uhag187)
Supplement: Supplementary file 1 [file web_material_uhag187.zip › FigS19.pdf]

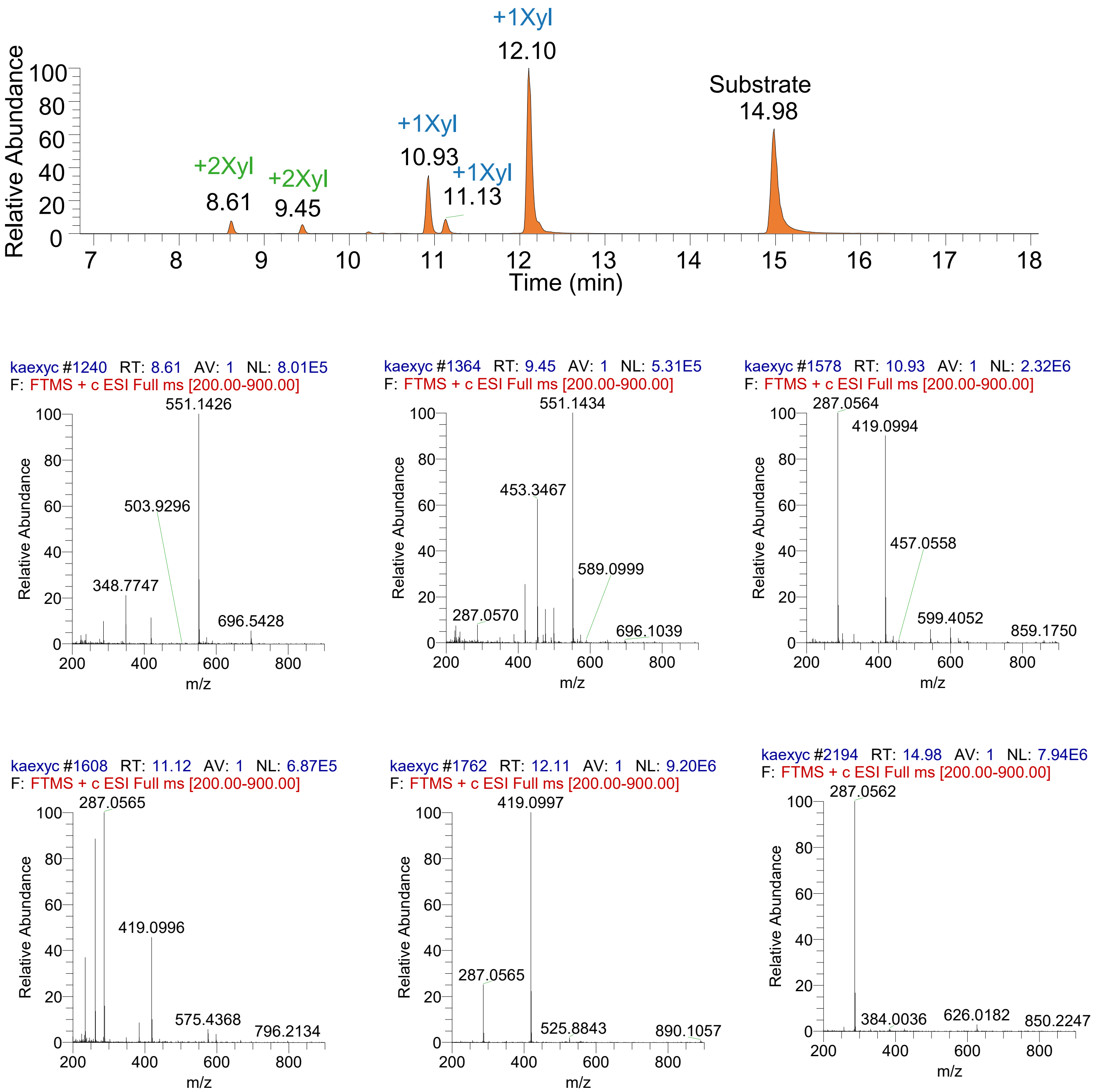
+1Xyl
12.10
Substrate
14.98
+1Xyl
10.93
+1Xyl
11.13
+2Xyl
8.61
+2Xyl
9.45
Relative Abundance
Time (min)
kaexyc #1240 RT: 8.61 AV: 1 NL: 8.01E5
F: FTMS + c ESI Full ms [200.00-900.00]
551.1426
503.9296
348.7747
696.5428
kaexyc #1364 RT: 9.45 AV: 1 NL: 5.31E5
F: FTMS + c ESI Full ms [200.00-900.00]
551.1434
453.3467
589.0999
287.0570
696.1039
kaexyc #1578 RT: 10.93 AV: 1 NL: 2.32E6
F: FTMS + c ESI Full ms [200.00-900.00]
287.0564
419.0994
457.0558
599.4052
859.1750
kaexyc #1608 RT: 11.12 AV: 1 NL: 6.87E5
F: FTMS + c ESI Full ms [200.00-900.00]
287.0565
419.0996
575.4368
796.2134
kaexyc #1762 RT: 12.11 AV: 1 NL: 9.20E6
F: FTMS + c ESI Full ms [200.00-900.00]
419.0997
287.0565
525.8843
890.1057
kaexyc #2194 RT: 14.98 AV: 1 NL: 7.94E6
F: FTMS + c ESI Full ms [200.00-900.00]
287.0562
384.0036
626.0182
850.2247
m/z
