# Supplementary material for: Spatial distribution of marker compounds in Epimedium sagittatum leaves and the role of UGT72B113 in generating kaempferol glycoside diversity
Source: Hortic Res. 2026 May 9;13(10):uhag187. doi: 10.1093/hr/uhag187 (PMC13610573; doi:10.1093/hr/uhag187)
Supplement: Supplementary file 1 [file web_material_uhag187.zip › FigS20.pdf]

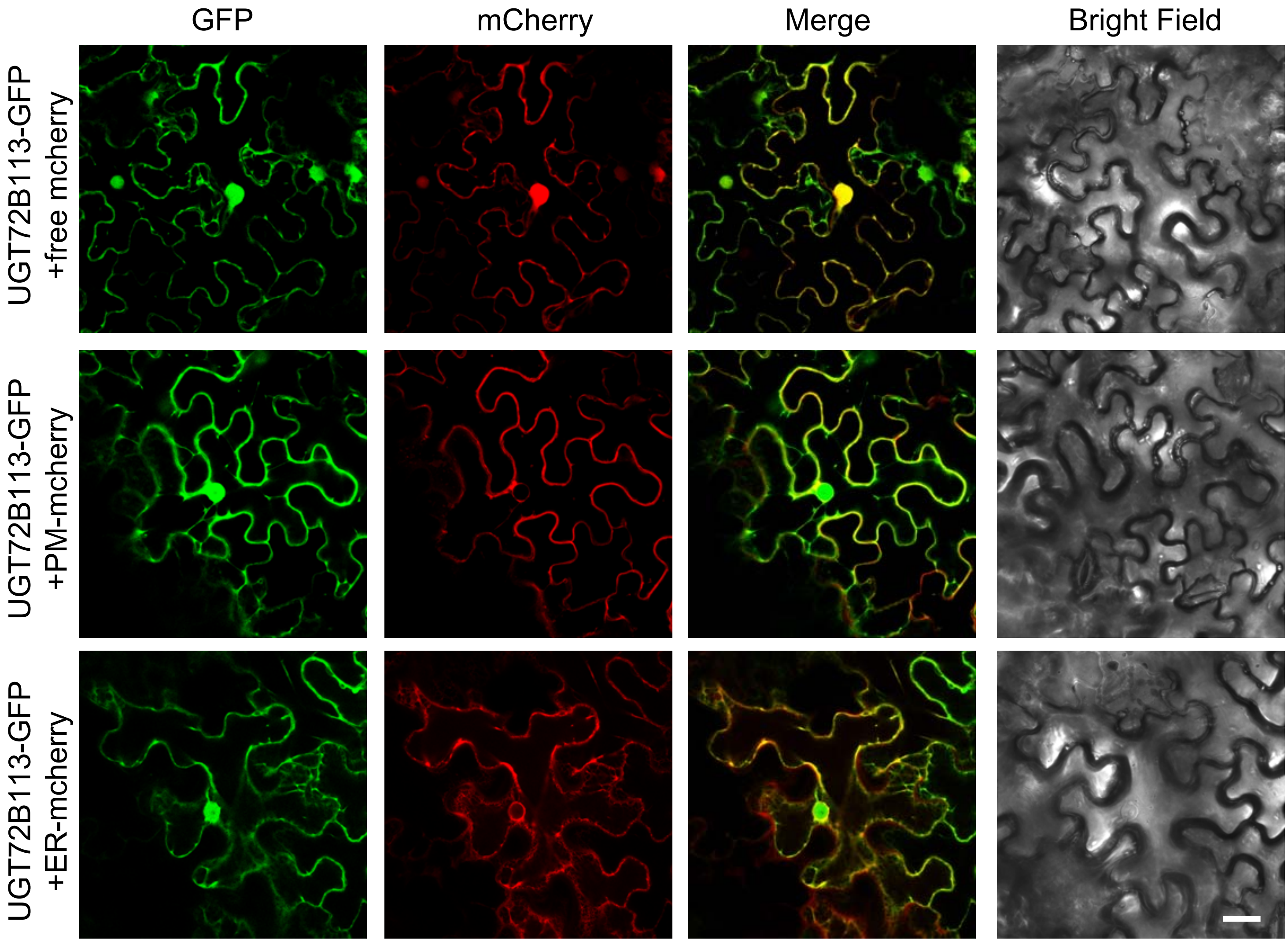
GFP
mCherry
Merge
Bright Field
UGT72B113-GFP
+free mcherry
UGT72B113-GFP
+PM-mcherry
UGT72B113-GFP
+ER-mcherry
