# Supplementary material for: Spatial distribution of marker compounds in Epimedium sagittatum leaves and the role of UGT72B113 in generating kaempferol glycoside diversity
Source: Hortic Res. 2026 May 9;13(10):uhag187. doi: 10.1093/hr/uhag187 (PMC13610573; doi:10.1093/hr/uhag187)
Supplement: Supplementary file 1 [file web_material_uhag187.zip › FigS21.pdf]

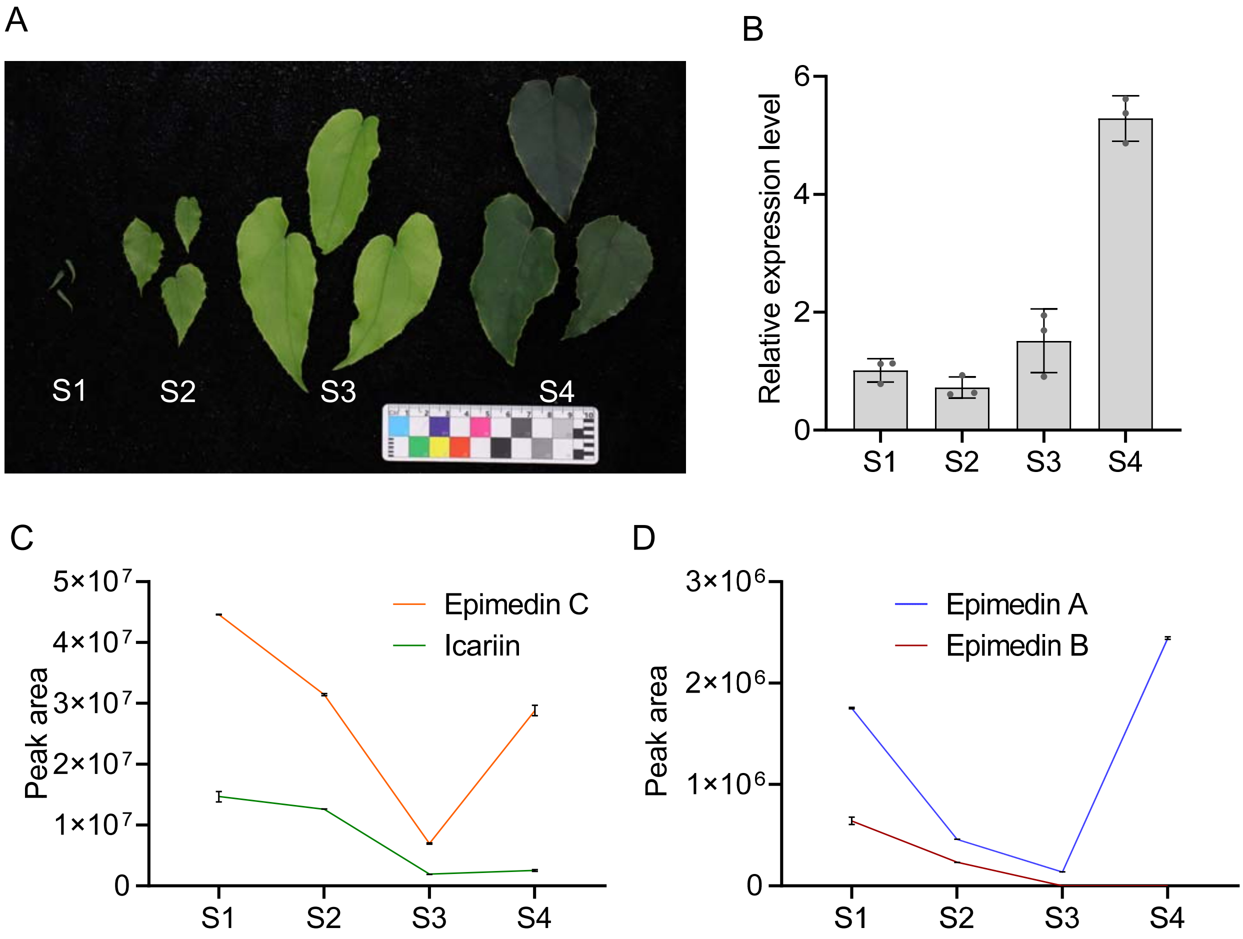
A
S1
S2
S3
S4
B
Relative expression level
0
2
4
6
S1
S2
S3
S4
C
Peak area
5×10⁷
4×10⁷
3×10⁷
2×10⁷
1×10⁷
0
Epimedin C
Icariin
S1
S2
S3
S4
D
Peak area
3×10⁶
2×10⁶
1×10⁶
0
Epimedin A
Epimedin B
S1
S2
S3
S4
