# Supplementary material for: Spatial distribution of marker compounds in Epimedium sagittatum leaves and the role of UGT72B113 in generating kaempferol glycoside diversity
Source: Hortic Res. 2026 May 9;13(10):uhag187. doi: 10.1093/hr/uhag187 (PMC13610573; doi:10.1093/hr/uhag187)

A

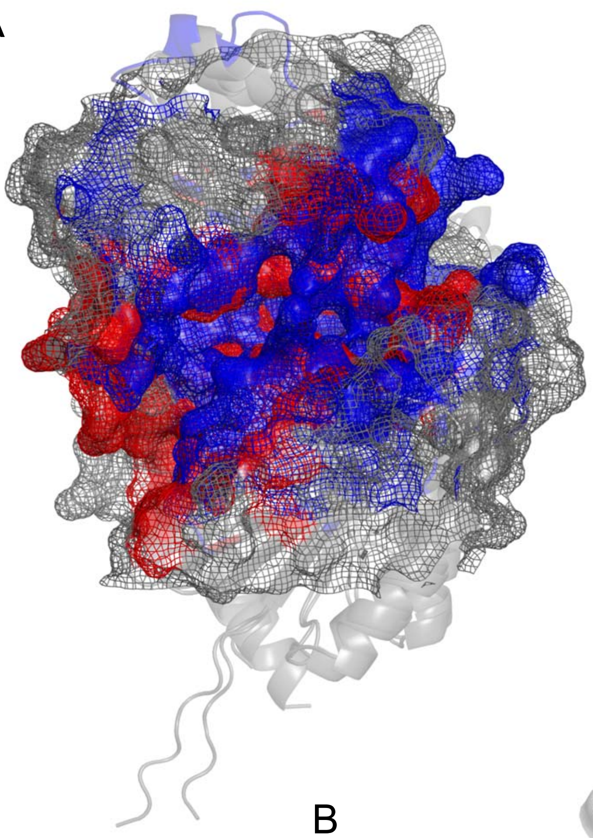

	Area(SA)(Å^2)	Volume (SA) (Å^3)
UGT84AA1	1560.985	1616.839
UGT72B113	1638.698	1358.029

B

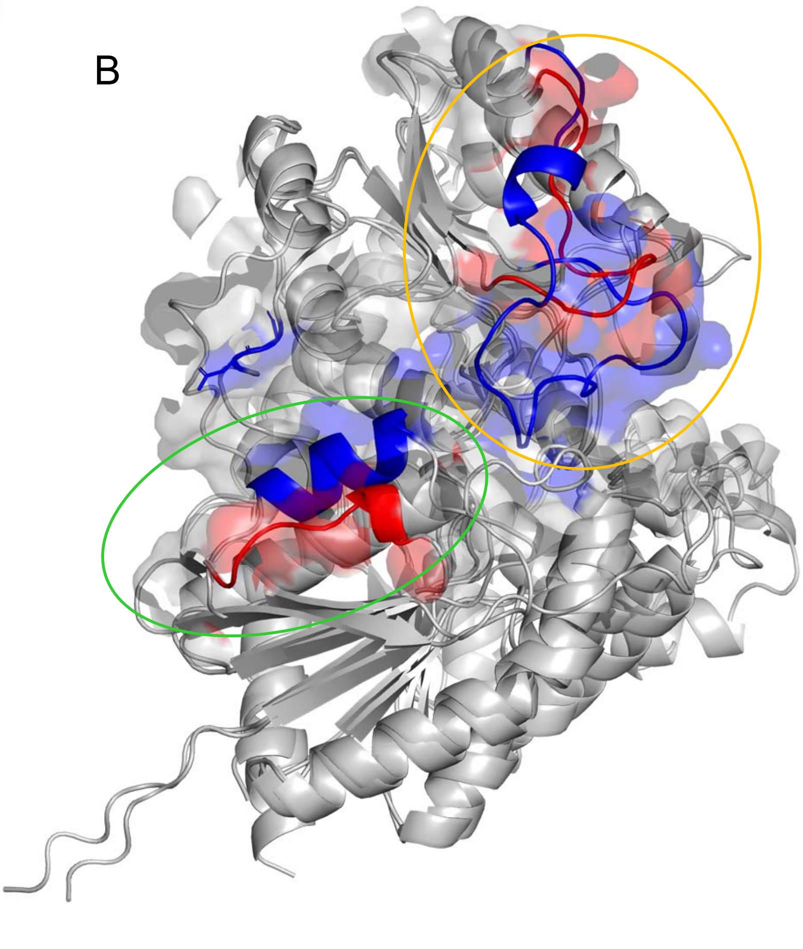

Supplement: Supplementary file 1 [file web_material_uhag187.zip › FigS22.pdf]
