# Supplementary material for: Spatial distribution of marker compounds in Epimedium sagittatum leaves and the role of UGT72B113 in generating kaempferol glycoside diversity
Source: Hortic Res. 2026 May 9;13(10):uhag187. doi: 10.1093/hr/uhag187 (PMC13610573; doi:10.1093/hr/uhag187)
Supplement: Supplementary file 1 [file web_material_uhag187.zip › Supplementary figures-revision1-nohighlighted.docx]

**Supplementary Figure 1. MALDI condition optimization using 9-AA as the matrix under negative ion mode.** (A) Total ion chromatogram of mixed standards. (B) Distributions of epimedin A, epimedin B, epimedin C, and icariin visualized by MSI with a 10-ppm mass tolerance. Signal intensity is represented by a green scale, with brighter green corresponding to higher intensity and black indicating no detectable signal.

**Supplementary Figure 2. MALDI condition optimization using SA as the matrix under negative ion mode.** (A) Total ion chromatogram of mixed standards. (B) Distributions of epimedin A, epimedin B, epimedin C, and icariin visualized by MSI with a 10-ppm mass tolerance. Signal intensity is represented by a green scale, with brighter green corresponding to higher intensity and black indicating no detectable signal.

**Supplementary Figure 3. MALDI condition optimization using SA as the matrix under positive ion mode.** (A) Total ion chromatogram of mixed standards. (B) Distributions of epimedin A, epimedin B, epimedin C, and icariin visualized by MSI with a 10-ppm mass tolerance. Signal intensity is represented by a green scale, with brighter green corresponding to higher intensity and black indicating no detectable signal.

**Supplementary Figure 4. MALDI condition optimization using CHCA as the matrix under negative ion mode.** (A) Total ion chromatogram of mixed standards. (B) Distributions of epimedin A, epimedin B, epimedin C, and icariin visualized by MSI with a 10-ppm mass tolerance. Signal intensity is represented by a green scale, with brighter green corresponding to higher intensity and black indicating no detectable signal.

**Supplementary Figure 5. MALDI condition optimization using CHCA as the matrix under positive ion mode.** (A) Total ion chromatogram of mixed standards. (B) Distributions of epimedin A, epimedin B, epimedin C, and icariin visualized by MSI with a 10-ppm mass tolerance. Signal intensity is represented by a green scale, with brighter green corresponding to higher intensity and black indicating no detectable signal.

**Supplementary Figure 6. MALDI condition optimization using 1,5-DAN as the matrix under negative ion mode.** (A) Total ion chromatogram of mixed standards. (B) Distributions of epimedin A, epimedin B, epimedin C, and icariin visualized by MSI with a 10-ppm mass tolerance. Signal intensity is represented by a green scale, with brighter green corresponding to higher intensity and black indicating no detectable signal.

**Supplementary Figure 7. MALDI condition optimization using 1,5-DAN as the matrix under positive ion mode.** (A) Total ion chromatogram of mixed standards. (B) Distributions of epimedin A, epimedin B, epimedin C, and icariin visualized by MSI with a 10-ppm mass tolerance. Signal intensity is represented by a green scale, with brighter green corresponding to higher intensity and black indicating no detectable signal.

**Supplementary Figure 8. Relative gene expression level of genes selected from flavonoid biosynthetic pathway.** Data are presented as mean ± SD.

**Supplementary Figure 9. Hierarchical clustering analysis of UGT family gene expression** **based on normalized RPKM values across all genes.** Candidate genes from this study were indicated in triangles.

**Supplementary Figure 10. SDS-PAGE analysis of recombinant protein expression.** Target protein bands are marked with red arrowheads. Lanes: EV, Empty vector control; 0, 0 mM IPTG induction; 1, 1 mM IPTG induction; A, auto‑induction medium; T, Total protein; S: Soluble protein; FT, Flow through part; W, Wash part; E: Elution part; PP: Final purified protein.

**Supplementary Figure 11. HPLC analysis of crude enzyme activity with kaempferol and kaempferide as substrates.** UDP‑glucose and UDP‑rhamnose are shown in orange and blue, respectively. Product peaks are indicated with red arrowheads.

**Supplementary Figure 12. HPLC analysis of crude enzyme activity with 8‑prenylkaempferol and icaritin as substrates.** UDP‑glucose and UDP‑rhamnose are shown in orange and blue, respectively. Product peaks are marked with red arrowheads.

**Supplementary Figure 13. HPLC analysis of crude enzyme activity with icariside I, icariside II, and icariin as substrates.** UDP‑glucose and UDP‑rhamnose are shown in orange and blue, respectively. Product peaks are indicated with red arrowheads.

**Supplementary Figure 14. Enzyme kinetic parameters for UGT72B113.** Data was shown in mean ± SD with three biological replicates. No data (n.d.) was got when using icaritin as substrate because the low concentration of the products.

**Supplementary Figure 15. UHPLC‑MS/MS analysis of the reaction catalyzed by UGT72B113 with kaempferol and UDP‑glucose.**

**Supplementary Figure 16. UHPLC‑MS/MS analysis of the reaction catalyzed by UGT72B113 with 8‑prenylkaempferol and UDP‑glucose.**

**Supplementary Figure 17. UHPLC‑MS/MS analysis of the reaction catalyzed by UGT72B113 with kaempferide and UDP‑glucose.**

**Supplementary Figure 18. UHPLC‑MS/MS analysis of the reaction catalyzed by UGT72B113 with icaritin and UDP‑glucose.**

**Supplementary Figure 19. UHPLC‑MS/MS analysis of the reaction catalyzed by UGT72B113 with kaempferol and UDP‑xylose.**

**Supplementary Figure 20. Subcellular localization of UGT72B113.** Confocal images of *Nicotiana benthamiana* leaf epidermal cells co-expressing UGT72B113-GFP with free mCherry (marker for nucleus and cytoplasm), PM-mCherry (plasma membrane marker), or ER-mCherry (endoplasmic reticulum marker). GFP fluorescence was excited at 488 nm and detected at 500–530 nm; mCherry fluorescence was excited at 561 nm and detected at 580–630 nm. All images share the same scale bar: 25 μm.

**Supplementary Figure 21. Relative gene expression level and metabolite accumulation during leaf development in *Epimedium sagittatum*.** (A) Phenotypes of leaves at five developmental stages. S1: folded young leaves, S2: expanded young leaves, S3: slightly leathery leaves, S4: leathery leaves. (B) Relative expression levels of *UGT72B113* in leaves at the indicated stages. (C) Relative content of the four marker compounds in leaves of different stages. The data in (B) and (C) are represented as means ± SD from three biological repeats.

**Supplementary Figure 22. Structural comparison of UGT84AA1 and UGT72B113.** (A) Volumetric differences of the active pockets. The substrate‑accessible cavities of UGT84AA1 (red) and UGT72B113 (blue) are shown as mesh and surface representations, with calculated solvent‑accessible area (SA) and volume (Vol). (B) Key structural features responsible for active‑pocket differences. The extended loop (W298‑L330) in UGT72B113 forms an occluding lid, whereas the shorter loop (W295‑L313) in UGT84AA1 maintains an open cavity (orange circle). A rigid α‑helix (S48‑L58) and protruding residues (N247, G248) in UGT72B113 constrict the entrance, in contrast to the flexible random coil (L45‑P52) in UGT84AA1 (green circle).
